# 3-Bromopyruvate-loaded bismuth sulfide nanospheres improve cancer treatment by synergizing radiotherapy with modulation of tumor metabolism

**DOI:** 10.1186/s12951-023-01970-8

**Published:** 2023-07-05

**Authors:** Yiman He, Huawan Chen, Wenbo Li, Lu Xu, Huan Yao, Yang Cao, Zhigang Wang, Liang Zhang, Dong Wang, Di Zhou

**Affiliations:** 1 Department of Ultrasound, the First Affiliated Hospital of Chongqing Medical University, Chongqing, 400042 P.R. China; 2 Department of Oncology, the First Affiliated Hospital of Chongqing Medical University, Chongqing, 400042 P.R. China; 3 Department of Nuclear Medicine, the First Affiliated Hospital of Chongqing Medical University, Chongqing, 400042 P.R. China; 4 Chongqing Key Laboratory of Ultrasound Molecular Imaging, Institute of Ultrasound Imaging, The Second Affiliated Hospital, Chongqing Medical University, Chongqing, 400010 P.R. China; 5 Department of Radiology, the First Affiliated Hospital of Chongqing Medical University, Chongqing, 400042 P.R. China

**Keywords:** Radiotherapy, 3-bromopyruvate, Bismuth (Bi) chalcogenides, Hypoxia relief, Pro-death autophagy

## Abstract

**Background:**

Radiotherapy (RT) is one of the most mainstream cancer therapeutic modalities. However, due to the lack of specificity of the radiation adopted, both normal and cancerous cells are destroyed indiscriminately. This highlights the crucial need to improve radiosensitization. This study aims to address this issue by constructing a multifunctional nanospheres that can sensitize multiple aspects of radiotherapy.

**Results:**

Nanospheres containing high atomic element Bi can effectively absorb ionizing radiation and can be used as radiosensitizers. Cell viability after Bi_2_S_3_ + X-ray treatment was half that of X-ray treatment alone. On the other hand, exposed 3-bromopyruvate (3BP) could reduce the overactive oxygen (O_2_) metabolism of tumor cells and alleviate tumor hypoxia, thereby promoting radiation-induced DNA damage. The combination index (CI) of 3BP and Bi_2_S_3_-based RT in Bi_2_S_3_-3BP + X-ray was determined to be 0.46 with the fraction affected (*f*_a_) was 0.5 via Chou-Talalay’s isobolographic method, which indicated synergistic effect of 3BP and Bi_2_S_3_-based RT after integration into Bi_2_S_3_-3BP + X-ray. Under the combined effect of 3BP and RT, autophagy was over-activated through starvation-induced and redox homeostasis dysregulation pathways, which in turn exhibited pro-death effects. In addition, the prepared nanospheres possess strong X-ray attenuation and high near-infrared (NIR) optical absorption, thus eliminating the need for additional functional components and could serve as bimodal contrast agents for computed tomography/photoacoustic (CT/PA) imaging.

**Conclusions:**

The rational design of multifunctional nanospheres with the unique properties provided a novel strategy to achieving high therapeutic efficacy in RT. This was accomplished through simultaneous activation of multiple sensitization pathways by increasing ionizing radiation, reducing tumor oxygen consumption, inducing pro-death autophagy, and providing multiple-imaging guidance/monitoring.

**Supplementary Information:**

The online version contains supplementary material available at 10.1186/s12951-023-01970-8.

## Introduction

Radiotherapy (RT), particularly external beam RT, is a mainstream cancer therapeutic modalities, with over 50% of cancer patients receiving it at some point during their illness [1, 2]. Generally, ionizing radiation induces cell death via two mechanisms - direct interaction with key cellular components to cause DNA damage or production of cytotoxic reactive oxygen species (ROS) [3, 4]. However, RT has certain limitations, such as unsatisfactory therapeutic efficiency with low X-ray does, and toxicity to the surrounding normal tissues associated with high intensity or multiple frequencies radiation beams [2, 5]. Therefore, there is an urgent need to introduce effective radiosensitization, which could improve curative effect of RT and reduce various side effects. Over the last few decades, nanospheres have emerged as promising tools to enhance the efficacy of RT, and have generated considerable interest. Nanospheres with high atomic number Z (e.g., iodine, gold, bismuth, gadolinium and platinum) have been reported to augment RT against targeted tissues by increasing secondary and oxyelectron production with relative low dose of X ray, thereby reducing the radiation damage to healthy normal tissues [5–7]. Bismuth (Bi) based nanomaterials are designed for use in RT due to their excellent X-ray attenuation capabilities [8]. Currently few nanoparticles are available on the market, which may be due to the difficulty of mass production with adaptive characteristics and the high cost of the entire process [6]. However, bismuth-containing composites or drugs have been extensively studied for their lower toxicity risk, good biocompatibility, cost effectiveness and longer blood circulation, making them an attractive candidate for radiosensitization [9]. Notably, porous bismuth sulfide (Bi_2_S_3_) nanospheres have attracted much attention for their ability to deliver good drugs [10].

Radioresistance is also associated with hypoxia in tumors, which can impair the efficacy of RT. Available oxygen can stabilize RT-induced DNA damage and produce stable organic peroxides that prevent DNA repair, thereby enhancing the degree of cellular damage [11]. In addition, RT-induced ROS generation capacity is closely related to the oxygen content of tumor tissue [12]. However, the poor vascularization, increased metabolic demand of the tumor cells, and increased interstitial pressure often led to hypoxia within solid tumors, resulting in resistance or failure of RT [13, 14]. Therefore, it is essential to develop effective strategies to alleviate tumor oxygen deprivation to improve the efficacy of RT. Strategies for mitigating the tumor hypoxia mainly include increasing oxygen supply and reducing oxygen consumption. Some studies have supplemented oxygen by using hydrogen peroxide (H_2_O_2_)-disintegrating materials or nanomaterials loaded with oxygen [15, 16]. However, nanomaterials used for decomposing H_2_O_2_ may suffer from low levels of H_2_O_2_ [17] or slow reactions with H_2_O_2_ [15]. Oxygen carriers (e.g., perfluorocarbons-based nanomaterials and oxygen-based microbubbles) also process some limitations, such as low oxygen loading [18] and rapid oxygen leakage [19]. These strategies may not prevent the development of tumor hypoxia due to the hyperactive oxygen metabolism prevalent in tumor cells, so it is particularly important to find ways to reduce oxygen consumption and thus alleviate the hypoxic microenvironment at its root [20].

3-bromopyruvate (3BP), an analogue of pyruvate and lactate, is a small molecule alkylating agent that alters tumor metabolism by inhibiting tumor cell glycolysis and mitochondrial oxidative phosphorylation (OXPHOS), which can significantly reduce the rate of intracellular oxygen consumption, effectively relieving tumor hypoxia and enhancing radiosensitization [21, 22].

Moreover, accumulating evidences have indicated that autophagy is activated under conditions of nutritional starvation and oxidative stress through highly regulated pathways associated with energy metabolism [23]. 3BP inhibits respiration, cuts off energy supply, and causes cell starvation, which can induce an increased level of cellular autophagy [24]. Additionally, the generation of ROS caused by ionizing radiation can also induce the occurrence of autophagy [25]. Autophagy is a catabolic process that degrades cytoplasmic proteins or organelles through the formation of autophagic lysosomes to achieve cell homeostasis and organelle renewal [26]. Based on the threshold effect of autophagy regulating cell death/survival, excessive activation of autophagy can promote cell death when it exceeds a certain threshold, and it may be more promising to play a positive role of autophagy in RT [27]. Under the combined effect of 3BP and RT, autophagy is over-activated through starvation-induced and redox homeostasis dysregulation pathways. Meanwhile, inhibition of aerobic glycolysis can directly induce apoptosis by reducing adenosine-triphosphate (ATP) levels in tumor cells [28]. As ATP is essential for supporting DNA replication and maintaining cell proliferation, inhibition of ATP can also enhance the efficacy of RT [28, 29]. However, the small molecular size of 3BP is difficult to retain in tumor tissue due to the enhanced permeability and retention (EPR) effect [30]. The past few decades have witnessed the evolution of nanomedicine from a biologically inert entity to a more intelligent system, aimed at improving in vivo functionality [31]. Therefore, the use of drug delivery vehicles such as mesoporous Bi_2_S_3_, to load 3BP is expected to overcome pathophysiological barriers to maximize efficacy.

**Scheme 1:**
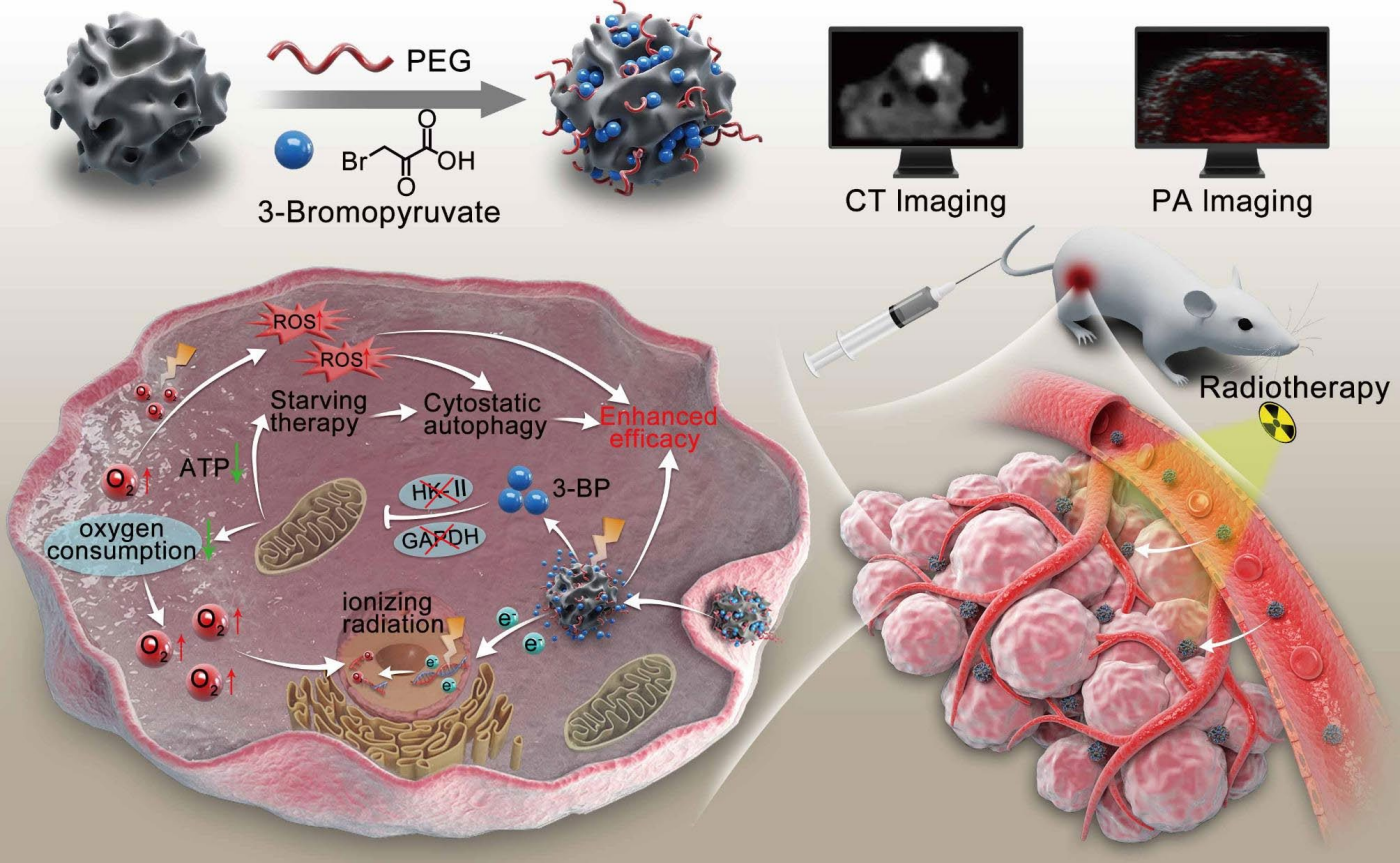
Schematic illustration of the synthesized Bi_2_S_3_-3BP nanospheres for effective radiosensitization through ionizing radiation enhancement, oxygen consumption reduction, pro-death autophagy activation, and CT/PA dual imaging monitoring/guidance

In this work, we rationally constructed multifunctional nanospheres (Bi_2_S_3_-3BP) for multiple RT sensitization by increasing the ionizing radiation effect through high atomic number nanospheres containing Bi, alleviating the tumor hypoxic microenvironment with 3BP, and co-activating cellular pro-death autophagy under nutrient starvation as well as oxidative stress conditions (Scheme 1). These Bi_2_S_3_-3BP nanospheres can effectively accumulate at the tumor site through EPR effect. The intertumoral enrichment can be monitored by CT/PA dual-modal imaging to formulate RT plans [32]. After X-ray irradiation, Bi_2_S_3_ in nanospheres induces DNA damage by directly absorbing ionizing radiation and generating large amounts of ROS [33]. While 3BP reduces oxygen consumption by inhibiting tumor cell metabolism, the relatively more sufficient oxygen to react with the broken ends of DNA to generate stable organic peroxides, which cannot be easily repaired and greatly enhance the degree of RT-induced cellular damage. Furthermore, 3BP and RT jointly triggered the occurrence of intracellular excessive autophagy, which showed a pro-death effect, further enhancing the efficacy of RT. The efficacy of radiosensitization by Bi_2_S_3_-3BP was systematically evaluated both in vitro and in vivo. By sensitizing RT in multiple ways, this work provides new insights to improve the accuracy and sensitivity of RT against tumor.

## Materials and methods

### Materials

All reagents used in this work were of analytical grade. Bismuth nitrate pentahydrate (Bi(NO_3_)_3_·5H_2_O, ≥ 99.99%), nitric acid (HNO_3_), sodium hydroxide (NaOH, ≥ 97.0%), polyvinylpyrrolidone (PVP, Mw ≈ 10,000 Da), ethylene glycol (EG, > 99%), thioacetamide (TAA, ≥ 99%), ethanol, acetone were purchased from Aladdin Reagent Co., Ltd. (China). Methoxy polyethylene glycol thiol (mPEG2K-SH, Mw ≈ 2000 Da) was purchased from Xi’an ruixi Biological Technology Co., Ltd (China). Cell Counting Kit-8 (CCK-8), 3-Methyladenine (3MA) and rapamycin (Rapa) were obtained from MedChemExpress (USA). 2-Deoxy-D-glucose (2DG) and rotenone were purchased from ApexBio Technology (USA). Enhanced ATP Assay Kit, Crystal Violet Staining Solution, Reactive Oxygen Species Assay Kit, BCA Protein Assay Kit, 4,6-Diamidino-2-phenylindole (DAPI) and 2,7-dichlorodihydrofluorescein diacetate (DCFH-DA) were purchased from Beyotime Biotechnology (China). Lactic Acid Content Test Kit, Glucokinase Activity Assay Kit, the monodansylcadaverine (MDC) staining kit, Triton X-100, bovine serum albumin and anti-fluorescence quenching agent were obtained from Beijing Solarbio Science & Technology Co., Ltd (China). 2.5% glutaraldehyde was obtained from Macklin (China). Spurr resin mixture was obtained from SPI-CHEM (USA). ROS-ID® Hypoxia/Oxidative stress detection kit was obtained from Enzo Life Sciences (USA). 3BP was purchased from Sigma-Aldrich (USA). Roswell Park Memorial Institute (RPMI)-1640 complete medium was purchased from Shanghai Zhong Qiao Xin Zhou Biotechnology Co., Ltd (China). Distilled water was obtained from a Millipore water system. All chemicals were of analytical grade and used without further purification.

### Synthesis of Bi_2_S_3_-3BP

To synthesis Bi_2_S_3_-3BP, 364 mg Bi(NO_3_)_3_·5H_2_O was dissolved in 10 mL HNO_3_ solution (1 mol/L). Then, 108 mg of NaOH, 1.2 g of PVP, and 50 mL of EG were added into the solution. After sufficiently mixing, the above mixture was transferred into a stainless-steel autoclave with Teflon liner, sealed, and maintained at 150 °C for 3 h. After cooling down to room temperature, the obtained milk-white product was precipitated by centrifugation and washed with distilled water. The Bi_2_O_3_ were utilized as the bismuth precursor and template to obtain highly porous Bi_2_S_3_ nanospheres.

Next, 100 mg of TAA was dissolved in 30 mL of distilled water, and then 10 mL of Bi_2_O_3_ suspension (in distilled water) was added. The mixture was then transferred into a stainless-steel autoclave, maintained at 150 °C for 12 h. The obtained black product was centrifuged, and then thoroughly washed with distilled water and absolute ethyl alcohol, respectively. The precipitate was dried in a vacuum oven at -50 °C.

For PEG coating, 10 mg of as-synthesized Bi_2_S_3_ product was dispersed in 10 mL absolute ethyl alcohol, and then 10 mg mPEG2K-SH was added. The mixture was subjected to rotary evaporation for 1 h. Excess mPEG2K-SH were removed by centrifugation and repeated washing using distilled water. The final Bi_2_S_3_ with PEG modification was resuspended in distilled water for further use. Next, 3BP was attached to the pores by magnetic stirring. In detail, 10 mL of Bi_2_S_3_ with PEG modification (1 mg/mL) and 30 mg 3BP were nixed and stirred overnight. Bi_2_S_3_-3BP was purified by centrifugation and repeated washing using distilled water.

### Characterization of Bi_2_S_3_-3BP

Transmission electron microscopy (TEM, Hitachi H-7600, Japan) was used to characterize the morphology of the sample. The crystalline form of the products was characterized based on X-ray diffraction (XRD) using an X-ray diffractometer (Panalytical X’ Pert PRO, Shimadzu, Japan) with Cu Kα radiation. X-ray photoelectron spectroscopy (XPS) was used to characterize the elemental composition of intermediate and final products using Thermo Scientific K-Alpha (Thermo Scientific, USA). Size distribution of the nanospheres was measured using dynamic light scattering (DLS, Malvern Instruments, Malvern, UK). The porosity of Bi_2_S_3_ nanospheres was analyzed by nitrogen adsorption–desorption isotherms (APSP 2460, Micromeritics, USA).

The infrared spectrum of Bi_2_S_3_, mPEG2K-SH and Bi_2_S_3_ with PEG modification were measured by using an FT-IR spectrometer (Nicolet 6700, Thermo Scientific, USA). The absorption spectra of Bi_2_S_3_ with PEG modification and Bi_2_S_3_-3BP (dissolved in deionized water) were determined with a UV-vis spectrophotometer (US-2550, Shimadzu, Japan). Different concentrations of free 3BP were used to obtain a calibration curve using UV-vis spectrometry, which was used to measure unloaded 3BP in the supernatant. Then the encapsulation efficiency and drug loading efficiency of Bi_2_S_3_-3BP can be calculated:

Encapsulation efficiency (EE) = (weight of loaded 3BP in Bi_2_S_3_-3BP / total weight of 3BP added) × 100%.

Drug loading efficiency (DLE) = (weight of loaded 3BP in Bi_2_S_3_-3BP / total weight of 3BP and Bi_2_S_3_ added) × 100%.

To investigate the drug release behavior of 3BP, Bi_2_S_3_-3BP (loaded 3BP: 6.8 mg) was dispersed in PBS (2.0 mL) and then stirred at 37 ° C. At a predetermined time points, the supernatant was extracted, then replaced with an equal volume of fresh PBS, the supernatant was collected, and the corresponding standard calibration curve was used to analyze the 3BP release.

### Cell culture and 4T1 tumor-bearing mice model

Murine breast cancer 4T1 cell lines were obtained from Procell Life Science & Technology Co., Ltd (China), and cultured in RPMI-1640 complete culture medium under a humidified atmosphere with 5% CO_2_ at 37 °C. All animal studies and procedures were conducted under a protocol approved by the Animal Ethics Committee of the First Affiliated Hospital of Chongqing Medical University (Review Lot No. 2021 − 717). All animal experiments were conducted in accordance with the guidelines of the Chongqing Management Approach of Laboratory Animal. All animals (female BALB/c mice with a weight of ≈ 20 g) were purchased from Hunan Slyke Jingda Laboratory Animal Co., Ltd (China). 4T1 tumor-bearing mice were established by subcutaneous administration of a cell suspension (1 × 10^6^ cells in 100 µL phosphate-buffered solution (PBS) for each mouse). The tumor size was measured using a vernier caliper, and tumor volumes were calculated as [0.5 × length × (width)^2^].

### Cellular uptake of Bi_2_S_3_-3BP

The cellular uptake of Bi_2_S_3_-3BP was investigated by laser scanning confocal microscopy (CLSM, Nikon Eclipse Ti, Tokyo, Japan) and flow cytometry (FACS, CytoFLEX Flow Cytometer, Becton Coulter, Brea, CA). Briefly, the 4T1 cells were seeded at a density of 1 × 10^5^ cells per CLSM dish and incubated for 24 h. Cells were further cultured in fresh RPMI-1640 medium containing FITC-labeled Bi_2_S_3_-3BP (Bi: 60 µg mL^− 1^) and incubated for 0, 1, 2, 3, 4 h. All cells were fixed with 4% formaldehyde (10 min), washed with PBS, and stained with DAPI (15 min) for CLSM observation. Intracellular uptake of Bi_2_S_3_-3BP at different time points was also quantified by flow cytometry.

### Detection of glycolysis inhibition after Bi_2_S_3_-3BP treatment

Tumor cells cultured in dishes were randomly divided into 6 groups (n = 3), and incubated with PBS, Bi_2_S_3_ (Bi: 60 µg mL^− 1^), Bi_2_S_3_-3BP (Bi: 60 µg mL^− 1^, 3BP: 10 µg mL^− 1^), 3BP (10 µg mL^− 1^), Rotenone (10 µg mL^− 1^), and 2DG (500 µg mL^− 1^) for 6 h, respectively. Western blot (WB) assay was performed to detect the expression of hexokinase-II (HK-II) and glyceraldehyde-3-phosphate dehydrogenase (GAPDH) in 4T1 cells after different treatments. Total protein was quantified using the BCA protein quantification kit. The detailed experimental procedure of WB is shown in the supplementary material. Tumor cells were divided into 3 groups (n = 3), and incubated with PBS, Bi_2_S_3_ (Bi: 60 µg mL^− 1^), and Bi_2_S_3_-3BP ((Bi: 60 µg mL^− 1^, 3BP: 10 µg mL^− 1^)) for 6 h, respectively. The viability of HK-II, intracellular lactate, and intracellular ATP were measured by the Glucokinase Activity Assay Kit, Lactic Acid Content Test Kit, and the Enhanced ATP Assay Kit according to standard protocols, respectively.

### Evaluation of hypoxia status in vitro after Bi_2_S_3_-3BP treatment

Tumor cells after adherence were incubated with control (untreated), Bi_2_S_3_ (Bi: 60 µg mL^− 1^) or Bi_2_S_3_-3BP (Bi: 60 µg mL^− 1^, 3BP: 10 µg mL^− 1^) for 6 h, the culture vessels were sealed with a sealant to prevent oxygen exchange. The dissolved oxygen in the medium before and after 6 h of the co-incubation was detected using the dissolved oxygen meter (B949712069, Mettler-Toledo Instruments Co., Ltd.). The intracellular hypoxia status was further detected with the ROS-ID® Hypoxia/Oxidative stress detection kit. Typically, cells were seeded in cell-culture dishes at a density of 1 × 10^5^ cells/dish, and cultured overnight. Then cells were cultured in serum-free RPMI-1640 medium containing either Bi_2_S_3_ (Bi: 60 µg mL^− 1^) or Bi_2_S_3_-3BP (Bi: 60 µg mL^− 1^, 3BP: 10 µg mL^− 1^) for another 6 h, and the system was sealed or not sealed with sealant. Cells treated with DFO, a chemical hypoxia inducer, were used as a positive control. All dishes were stained with hypoxia red detection reagent for 30 min. After washing with PBS, the dishes were observed by CLSM.

### In vitro anti-tumor efficacy

To estimate cytotoxicity in vitro, 4T1 cells were seeded in 96-well plates, and were cocultured with media containing different concentrations of Bi_2_S_3_ or Bi_2_S_3_-3BP for 6 h, and exposed to X-ray irradiation (A Linear accelerator (VARIAN, 21EX) was used. Cells/mice were placed 100 cm from the X-ray source. Energy of radiation was 6 MV.). Cell viability was determined by a CCK-8 method. Chou-Talalay’s isobolographic method [34] was calculated for Bi_2_S_3_-based RT and 3BP on Bi_2_S_3_-3BP + X-ray using the equation combination index (CI) = (*C*_BIS(+3BP)_/*C*_BIS_) + (*C*_3BP(+BIS)_/*C*_3BP_) + α[(*C*_BIS(+3BP)_)(*C*_3BP(+BIS)_)/(*C*_BIS_)(*C*_3BP_)], where *C* is the concentration of BIS (Bi_2_S_3_ + X-ray) and 3BP (Bi_2_S_3_-3BP) alone or in combination (Bi_2_S_3_-3BP + X-ray) at a given molar ratio to obtain a given *f*a. The effect fraction (*f*a) is the cell death measured by the CCK8 method. Using the more conservative reciprocal assumption (α = 0), CI < 1, CI = 1 and CI > 1 indicate synergistic, additive and antagonistic effects, respectively. For the detection of intracellular ROS generation. Tumor cells cultured in dishes were randomly divided into 5 groups, including control (untreated), X-ray (only), Bi_2_S_3_-3BP (Bi: 60 µg mL^− 1^, 3BP: 10 µg mL^− 1^), Bi_2_S_3_ (Bi: 60 µg mL^− 1^) + X‑ray, and Bi_2_S_3_-3BP (Bi: 60 µg mL^− 1^, 3BP: 10 µg mL^− 1^) + X‑ray. Intracellular ROS generation after different treatments were detected by CLSM after DCFH-DA staining.

Cloning experiments and DNA damage examination were further conducted under anaerobic and aerobic conditions, respectively. 4T1 cells were seeded into 6-well plates at a density of 6000 cells per well and divided into 3 groups including control (untreated), Bi_2_S_3_ (Bi: 60 µg mL^− 1^), and Bi_2_S_3_-3BP (Bi: 60 µg mL^− 1^, 3BP: 10 µg mL^− 1^). All groups received X-ray. Next, fresh RPMI-1640 medium was used to culture cells for additional 7 days. Finally, cells were rinsed with PBS, fixed by 4% paraformaldehyde, and stained with crystal violet about 30 min. Cell colonies were then counted. As for DNA damage assessment by γ‑H2AX immunofluorescence staining, 1 h after X‑ray exposure, the cells were washed with PBS, fixed by 4% formaldehyde, permeabilized with PBS containing 0.5% (v/v) Triton X-100, treated with 5% bovine serum albumin, and incubated with γ‑H2AX (Rabbit monoclonal antibody, 1:250, Abcam) primary antibody at 4 °C overnight. After washing with PBS, cells were incubated with Rhodamine (TRITC)–Conjugated Goat anti-rabbit IgG secondary antibody (1:100, ZSGB-BIO, Beijing, China) at 37 °C for 1 h, and stained with DAPI for CLSM observation. The positive area of DNA damage in every sample was analyzed by ImageJ software.

### Detection of cellular autophagy

Expression of autophagy-related proteins P62 and LC3 was detected by Western blot assay. Besides, immunofluorescence staining was used to detect the formation of LC3 dots. Cytotoxicity of autophagy inhibitor/promoter was detected by CCK-8 kit. The details were described in the supplementary materials. Autophagosomes formed during autophagy were stained with the MDC staining kit, 4T1 cells were seeded in CLSM dishes for 4 groups: Control (untreated), Bi_2_S_3_-3BP (Bi: 60 µg mL^− 1^, 3BP: 10 µg mL^− 1^), Bi_2_S_3_ (Bi: 60 µg mL^− 1^) + X-ray, and Bi_2_S_3_-3BP (Bi: 60 µg mL^− 1^, 3BP: 10 µg mL^− 1^) + X-ray, respectively. Then 4T1 cells were incubated with MDC (Solarbio, G0170) according to the specification for 30 min. After washed by PBS for three times, the autophagosomes dots were monitored by CLSM.

Autophagosomes were observed more intuitively by TEM, 4T1 cells were treated with Control (untreated), Bi_2_S_3_-3BP (Bi: 60 µg mL^− 1^, 3BP: 10 µg mL^− 1^), Bi_2_S_3_ (Bi: 60 µg mL^− 1^) + X-ray, and Bi_2_S_3_-3BP (Bi: 60 µg mL^− 1^, 3BP: 10 µg mL^− 1^) + X-ray, respectively. Then 4T1 cells were fixed with 2.5% glutaraldehyde for more than 4 h. After washed by PBS for three times, 4T1 cells were fixed by osmium tetroxide for 2 h, dehydrated with graded ethanol and transferred to absolute acetone for 20 min, then embedded in Spurr resin mixture to form ultrathin sections. Finally, the autophagosomes in these cell ultrathin sections were detected by TEM.

For the detection of cytotoxicity of RT with autophagy inhibitor/promoter, 4T1 cells were seeded in a 96-well plate and treated as follows: Bi_2_S_3_ (Bi: 60 µg mL^− 1^) + RAPA (3.5 µg mL^− 1^), Bi_2_S_3_ (Bi: 60 µg mL^− 1^) + 3-MA (6 µg mL^− 1^), Bi_2_S_3_-3BP (Bi: 60 µg mL^− 1^, 3BP: 10 µg mL^− 1^), Bi_2_S_3_-3BP (Bi: 60 µg mL^− 1^, 3BP: 10 µg mL^− 1^) + RAPA (3.5 µg mL^− 1^), and Bi_2_S_3_-3BP (Bi: 60 µg mL^− 1^, 3BP: 10 µg mL^− 1^) + 3-MA (6 µg mL^− 1^), respectively. Cells were incubated with the corresponding nanospheres and drug for 6 h, and then exposed to X-ray. After 12 h incubation, CCK-8 kit were used to detect the cell viabilities.

### Evaluation of tumor hypoxia relief and autophagy in vivo

Tumor oxyhemoglobin levels were assessed by PA imaging in an oxyhem mode. After post-injection with Bi_2_S_3_-3BP (Bi: 5 mg mL^− 1^,3BP: 0.8 mg mL^− 1^, 200 µL) and saline, oxyhemoglobin saturation (sO_2_ Avr Total) in the tumor at different time points (0, 2, 4, 6 and 12 h) were determined. Moreover, intertumoral hypoxia status was evaluated by immunofluorescence staining. 4T1 tumor-bearing mice were assigned into 3 groups (6 mice per group) as follows: (1) Control (200 µL of saline injection), (2) Bi_2_S_3_ (Bi: 5 mg mL^− 1^, 200 µL), and (3) Bi_2_S_3_-3BP (Bi: 5 mg mL^− 1^,3BP: 0.8 mg mL^− 1^, 200 µL). The mice were intravenously injected with corresponding nanospheres. After 6 h of the injection, 3 mice in each group were sacrificed, and the tumor tissues were harvested for hypoxia-inducible factor 1 (HIF-1α) immunofluorescence staining. The other 3 mice in each group were intravenously injected with pimonidazole hydrochloride (60 mg kg^− 1^) (Hypoxyprobe-1 plus kit, Hypoxyprobe Inc.). After almost an hour and a half, tumor tissues were harvested for the detection of Pimonidazole. The fluorescence of HIF-1α and pimonidazole was observed by CLSM and the average fluorescence intensity was analyzed by ImageJ software.

In vivo ROS and autophagy levels of tumor tissue after different treatments were evaluated by immunofluorescence staining. 4T1 tumor-bearing mice were assigned into 6 groups (6 mice in each group) as follows: (1) Control (200 µL of saline injection), (2) Bi_2_S_3_ (Bi: 5 mg mL^− 1^, 200 µL), (3) Bi_2_S_3_-3BP (Bi: 5 mg mL^− 1^,3BP: 0.8 mg mL^− 1^, 200 µL), (4) X-ray, (5) Bi_2_S_3_ (Bi: 5 mg mL^− 1^, 200 µL) + X-ray and (6) Bi_2_S_3_-3BP (Bi: 5 mg mL^− 1^,3BP: 0.8 mg mL^− 1^, 200 µL) + X-ray. The mice were intravenously injected with corresponding nanospheres. After 6 h, the mice in X-ray, Bi_2_S_3_ + X-ray and Bi_2_S_3_-3BP + X-ray groups were exposed to X-ray. Then 3 mice in each group were intravenously injected with DCFH-DA (10 µM, 200 µL). After 60 min, these mice were sacrificed and tumor tissues were harvested by frozen tumor slides for detecting ROS. Other 3 mice in each group were executed and the tumor tissues were harvested for detecting LC3 (autophagy level) by immunofluorescence staining.

### Anti-tumor efficacy in vivo

The tumor-bearing mice were randomly divided into six groups (5 mice in each group) as follows: (1) Control (200 µL of saline injection), (2) Bi_2_S_3_ (Bi: 5 mg mL^− 1^, 200 µL), (3) Bi_2_S_3_-3BP (Bi: 5 mg mL^− 1^,3BP: 0.8 mg mL^− 1^, 200 µL), (4) X-ray, (5) Bi_2_S_3_ (Bi: 5 mg mL^− 1^, 200 µL) + X-ray and (6) Bi_2_S_3_-3BP (Bi: 5 mg mL^− 1^,3BP: 0.8 mg mL^− 1^, 200 µL) + X-ray. The mice were intravenously injected with the corresponding nanospheres on day 0 and day 4, respectively. Mice in X-ray, Bi_2_S_3_ + X-ray, and Bi_2_S_3_-3BP + X-ray groups received X-ray irradiations at 6 h after the injection. Their body weight and tumor diameter were recorded every other day during the 14 days of observation. The tumor volumes were calculated as [0.5 × length × (width)^2^]. On day 14, the mice were sacrificed, and the tumors from each group were harvested and photographed. Tumor tissues from each group were fixed in 4% paraformaldehyde for histological analysis, including hematoxylin & eosin (H&E), proliferating cell nuclear antigen (PCNA) and terminal deoxynucleotidyl transferase-mediated dUTP-biotin nick end labeling (TUNEL) assays. The mean fluorescence intensity was analyzed by ImageJ software.

### Statistical analysis

All data were expressed as mean ± standard deviation (SD). The GraphPad Prism 8.0 software was used for statistical analysis. Two-tailed Student’s *t*-test and one-way ANOVA test were used to detect statistical differences between two groups and multiple groups, respectively. p-Values are indicated as n.s. = p > 0.05, *p < 0.05; **p < 0.01; ***p < 0.001, respectively.

## Result and discussion

### Synthesis and characterization of Bi_2_S_3_-3BP

**Fig. 1:**
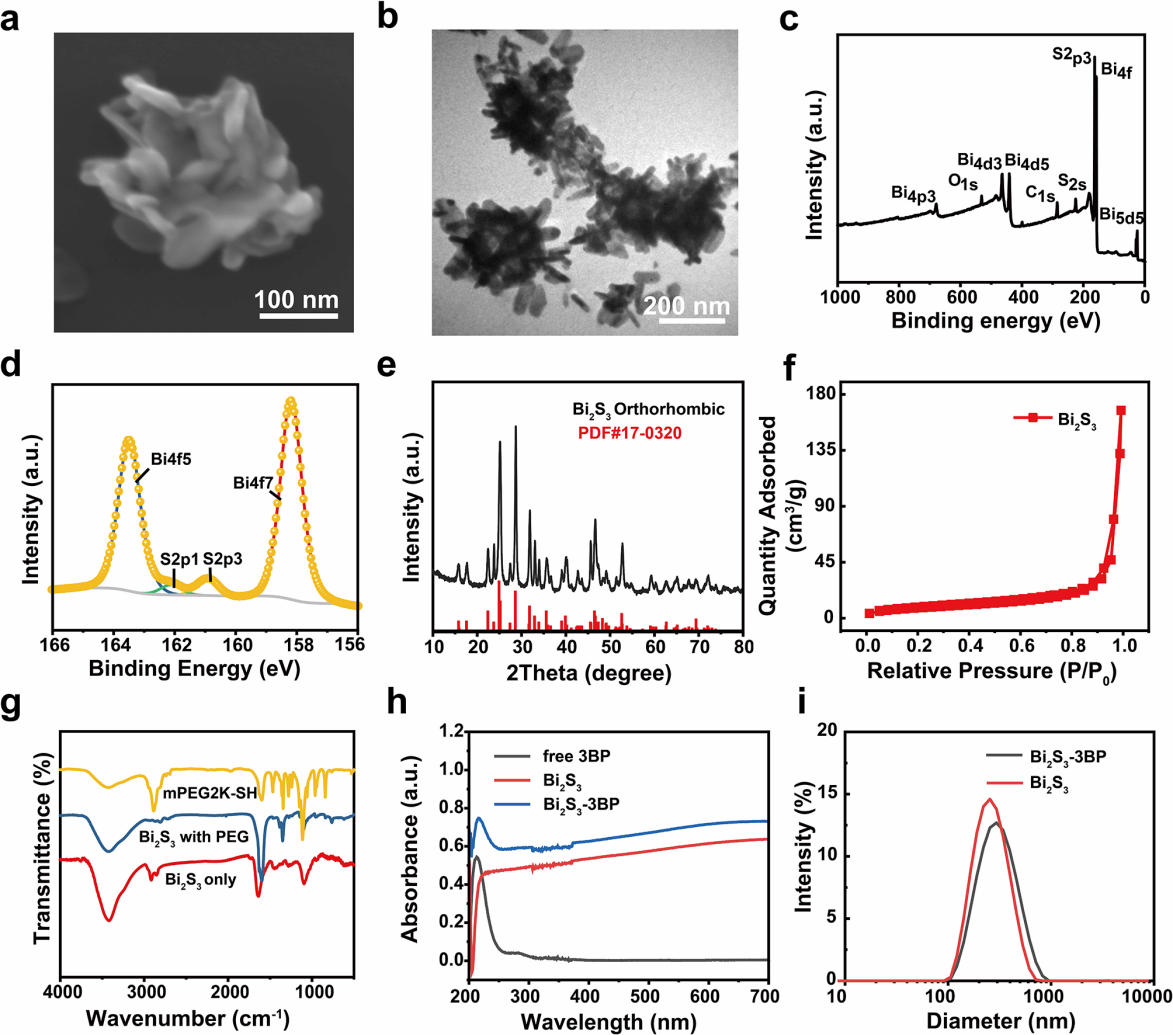
Synthesis and characterization of Bi_2_S_3_-3BP. **(a)** SEM image of Bi_2_S_3_. **(b)** TEM image of Bi_2_S_3_. **(c)** XPS survey spectrum of Bi_2_S_3_. **(d)** XPS spectra of Bi 4f, and S 2p orbits of Bi_2_S_3_. **(e)** XRD patterns of Bi_2_S_3_. **(f)** N_2_ adsorption-desorption isotherm of Bi_2_S_3_ nanospheres. **(g)** FT-IR spectra of mPEG2K-SH, Bi_2_S_3_ with PEG modification, and Bi_2_S_3_. **(h)** Absorbance spectra of free 3BP, Bi_2_S_3_ with PEG modification, and Bi_2_S_3_-3BP as recorded by a UV–vis spectrophotometer. **(i)** Size distribution by intensity of Bi_2_S_3_ and Bi_2_S_3_-3BP as measured by DLS.

To fabricate 3-BP loaded nanospheres, we first synthesized Bi_2_S_3_ nanospheres by a facile and effective hydrothermal method, using Bi_2_O_3_ as the bismuth precursor. To improve the dispersion and optimize the colloidal stability of Bi_2_S_3_ nanospheres, we stably attached mPEG2K-SH to the surface of Bi_2_S_3_ nanospheres by rotary evaporation. Next, 3BP was loaded into the pore by magnetic stirring to obtain Bi_2_S_3_-3BP (Scheme 1). As shown in Figure S1-S2, the Bi_2_O_3_ nanoparticles, bismuth precursor, revealed a spherical morphology. The obtained Bi_2_S_3_ nanospheres showed a distinct star-shaped structure (Fig. 1a and b). The elemental composition of Bi_2_S_3_ nanospheres was analyzed by XPS. The full spectrum scanning results showed that Bi_2_S_3_ nanospheres primarily contained Bi, S, O and C elements (Fig. 1c). Bi 4f7 and S 2p3 correspond to Bi^3+^ (158.2 eV) and S^2−^ (160.9 eV), respectively, providing direct evidence for the successful synthesis of Bi_2_S_3_ (Fig. 1d). XRD further showed that the representative diffraction peaks of Bi_2_S_3_ nanospheres aligned well with the standard pattern of Bi_2_S_3_ (JCPDS Card No. 17–0320), indicating the successful preparation of Bi_2_S_2_ crystals (Fig. 1e). The porosity of Bi_2_S_3_ was studied by Brunauer-Emmet-Teller (BET) analysis of nitrogen adsorption-desorption isotherm. Bi_2_S_3_ exhibit type-IV isotherms, whose hysteresis curves correspond to mesoporous/microporous structures (Fig. 1f**)**. The BET specific surface area of Bi_2_S_3_ was ~ 30.31 m^2^g^− 1^. In addition, the pore size distribution shows that Bi_2_S_3_ is dominated by mesoporous pores of 2 to 4 nm (Figure S3). FT-IR was used to confirm the successful surface modification of PEG (Fig. 1g). Comparing the spectra of the three materials, it was found that the characteristic peaks of Bi_2_S_3_ with PEG modification samples were from both those of mPEG2K-SH and Bi_2_S_3_, indicating the successful conjugation of mPEG2K-SH onto Bi_2_S_3_. The obtained Bi_2_S_3_ with PEG modification exhibited good stability and dispersion in various physiological conditions (Figure S4-S5). UV-Vis spectra (Fig. 1h) confirmed the successful loading of 3BP, the Bi_2_S_3_-3BP showed a broad absorption peak at ~ 212 nm compared with bare Bi_2_S_3_, which was corresponding to 3BP. The wavelength at 212 nm is too close to the initial wavelength of UV–vis spectrum measurement of 200 nm, the absorbance peak here showed red-shifted, therefore a calibration curve was obtained at 282 nm (Figure S6). According to the UV-Vis absorbance results, the EE of 3BP in Bi_2_S_3_-3BP were calculated to be 22.9 ± 1.6% and and DLE for 3BP was determined as 17.2 ± 1.2%, respectively. To examine drug release properties, Bi_2_S_3_-3BP solutions were investigated. The results showed that 58.1% of 3BP was released after 18 h (Figure S7). The average size of Bi_2_S_3_-3BP was about 260 nm as measured by DLS (Fig. 1i).

### Cellular uptake of Bi_2_S_3_-3BP and inhibition of glycolysis

**Fig. 2:**
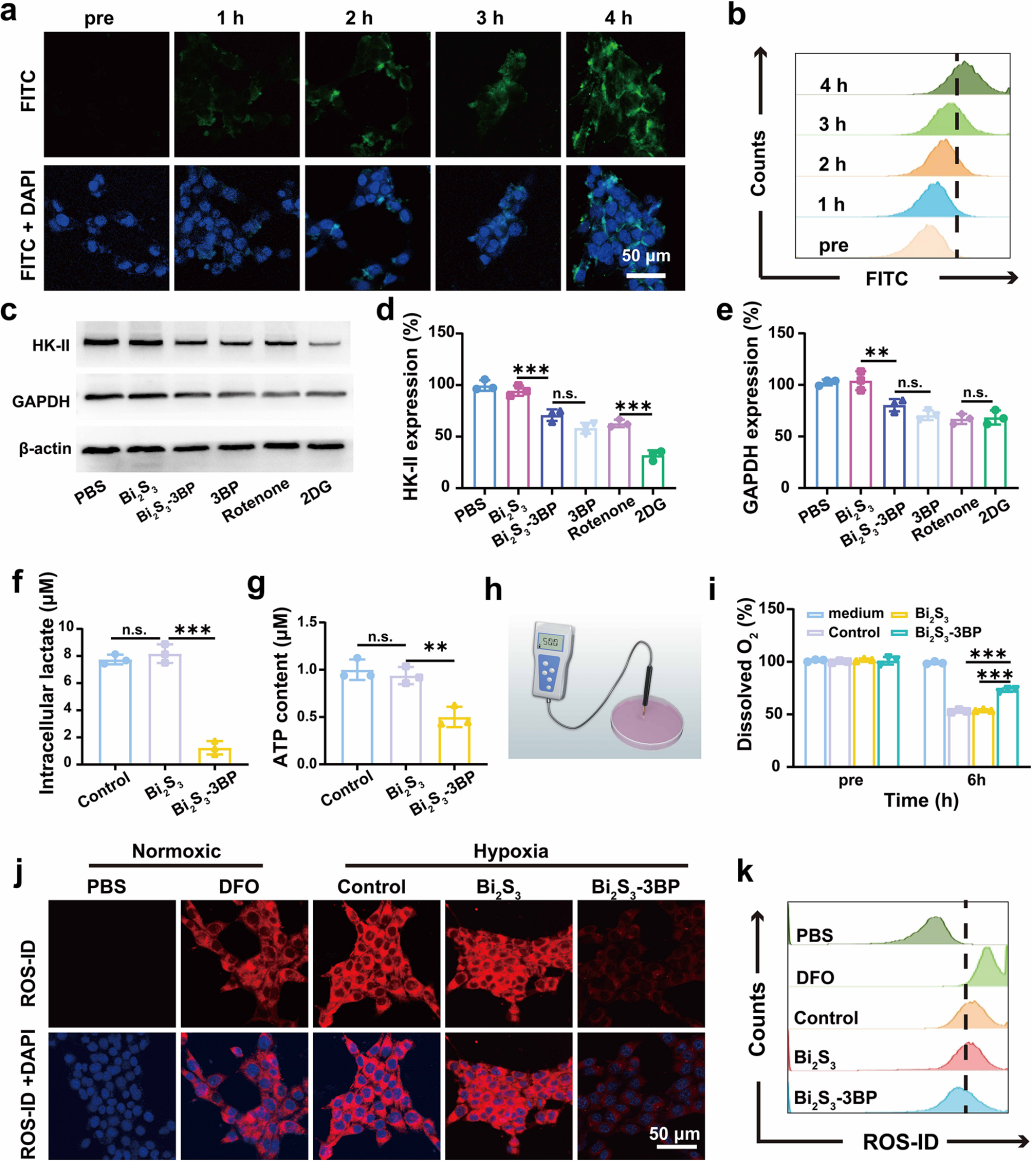
Cellular uptake of Bi_2_S_3_-3BP and inhibition of glycolysis. **(a-b)** Intracellular uptake of Bi_2_S_3_-3BP (labeled with FITC) observed by CLSM and quantified by flow cytometry analysis. **(c)** HK-II and GAPDH expression measured by western blot. **(d-e)** Quantitative analysis of relative HK-II and GAPDH expression (n = 3). **(f)** Intracellular lactate levels (n = 3). **(g)** Intracellular ATP levels (n = 3). **(h)** Schematic illustration about measuring the O_2_ consumption. **(i)** Relative dissolved oxygen (D.O.) changes in the cell media of different groups (n = 3). **(j-k)** Cells stained with ROS-ID in different groups observed by CLSM and quantified by flow cytometry. n.s.: no significance, *P < 0.05, **P < 0.01, ***P < 0.001

Efficient cellular uptake is critical for the subsequent therapeutic approaches. The cellular uptake of Bi_2_S_3_-3BP nanospheres was investigated by CLSM and flow cytometry analysis. In a prolonged observation, increasing green fluorescence intensity was observed in 4T1 cells after 4 h of co-culture (Fig. 2a), which can be assigned to Bi_2_S_3_-3BP (labeled with FITC). The effective uptake is also substantiated by flow cytometry results (Fig. 2b). These results indicate a potentially efficient accumulation of Bi_2_S_3_-3BP in tumor cells.

Tumor hypoxia severely hampers the therapeutic efficacy of RT. Regulating the metabolism of tumor cells is an emerging approach to alleviate hypoxia [35] In this study, we introduced 3BP to regulate cellular respiratory metabolism. Considering that HK-II and GAPDH are key enzymes involved in glycolysis and OXPHOS [22], a western blot analysis was performed to detect the expression of these two enzymes in 4T1 cells after various treatments. A typical glycolysis inhibitor (2DG) and oxidative phosphorylation inhibitor (Rotenone) were used as controls. Bi_2_S_3_ without 3BP loading had no significant effect on the expression levels of HK-II and GAPDH. When 4T1 cells were incubated with Bi_2_S_3_-3BP and free 3BP, the expression levels of HK-II decreased by 23.6% and 36.3% (Fig. 2c-d), demonstrating the function of 3BP. Meanwhile, HK-II viability was also significantly reduced by 33.5% after Bi_2_S_3_-3BP treatment (Figure S8). The expression levels of GAPDH also decreased by 23.7% and 33.5%, correspondingly (Fig. 2c and e). Therefore, it can be seen that 3BP played a key role in the inhibition of HK-II and GAPDH, and the inhibitory capacity is comparable to that of 2DG, a typical glycolysis inhibitor. When the glycolytic pathway was inhibited, lactate, the end product of glycolysis, decreased. As expected, the intracellular lactate concentration decreased from 7.7 µM to 1.2 µM after Bi_2_S_3_-3BP treatment (Fig. 2f). In contrast, Bi_2_S_3_ without 3BP did not affect intracellular lactate production. Aerobic glycolysis, also known as the Warburg effect, has been widely documented as the main energy source in most cancer cells [36]. In this study, 3BP disrupted the energy supply by inhibiting glycolysis, which can affect a series of biological events, as ATP is required for numerous biological processes, such as DNA replication and transcription [37]. ATP levels of 4T1 cells after different treatments were measured quantitatively. Bi_2_S_3_ barely affected the intracellular ATP level. When 4T1 cells were incubated with Bi_2_S_3_-3BP, the intracellular ATP level decreased to 50.1% (Fig. 2g), promoting tumor inhibition.

In addition to inhibiting glycolysis, 3BP also inhibits mitochondrial OXPHOS, resulting in a significant reduction in intracellular oxygen consumption. Therefore, oxygen consumption in the cell culture media was monitored by measuring changes in dissolved oxygen (D.O.) (Fig. 2h-i). After 6 h treatment with PBS or Bi_2_S_3_, compared with the untreated group, the D.O. decreased sharply to 53.5% and 54.7%, respectively. The D.O. of Bi_2_S_3_-3BP treatment group dropped much less, which was only 26.9%. (Fig. 2j). These results indicated that Bi_2_S_3_-3BP could effectively reduce oxygen consumption in cancer cells and potentially counteract hypoxia. To verify this, intracellular hypoxia level was monitored with a hypoxia detection kit. Under the hypoxia condition, both Bi_2_S_3_-treated cells and untreated cells showed intense red fluorescence similar to that of DFO-treated cells (positive control) (Fig. 2i), whereas the red fluorescence intensity of Bi_2_S_3_-3BP-treated cells was significantly reduced. These results are consistent with the flow cytometry analysis (Fig. 2k) and fluorescence quantification (Figure S9) obtained from ImageJ. These results confirmed that 3BP-containing nanospheres could effectively reduce the consumption of oxygen and alleviate intracellular hypoxia, which would improve the efficacy of RT.

### Synergistic effect of Bi_2_S_3_-3BP on in vitro RT

**Fig. 3:**
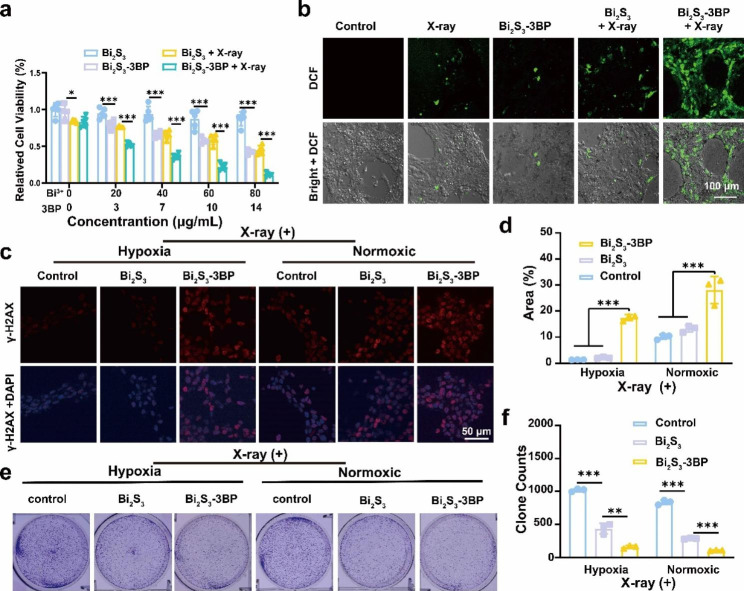
In vitro RT effect of Bi_2_S_3_-3BP. **(a)** Cell viability after different treatments (n = 5). **(b)** Intracellular ROS level observed by CLSM after different treatments. **(c)** DNA damage staining after different treatments. Red: γ-H2AX signal (dsDNA damage staining); blue: DAPI (nuclear staining). **(d)** Statistics of DNA damage staining in **(c)** (n = 3). **(e-f)** Images of 4T1 cell clones after different treatments and the corresponding quantification (n = 3). *P < 0.05, **P < 0.01, ***P < 0.001

The efficacy of RT was positively correlated with oxygen levels, which can prevent repair of DNA damage and thus achieve radiosensitization. After verifying that Bi_2_S_3_-3BP can effectively alleviate hypoxia in vitro, we next evaluated the effect of Bi_2_S_3_-3BP on radiosensitization at the cellular level. In the absence of X-ray irradiation, Bi_2_S_3_ did not exhibit any cytotoxicity even when the concentration reached 80 µg mL^− 1^. X-ray alone exhibited only slight ionizing radiation toxicity, while Bi_2_S_3_ + X-ray showed a gradual increase in toxicity with elevated concentrations of Bi_2_S_3_, which should be ascribed to the intrinsically enhanced ionizing radiation of Bi. Meanwhile, Bi_2_S_3_-3BP exhibited significant cytotoxicity in the absence of radiation, with a cell viability of 42.4%, which could be attributed to the cell growth inhibition of 3BP. In contrast, Bi_2_S_3_-3BP + X-ray could further reduce cell activity, with a cell viability of only 11.2% (Fig. 3a). And the therapeutic effect of the combined treatment (Bi_2_S_3_-3BP + X-ray) presented much stronger cell growth inhibition than that of Bi_2_S_3_-based RT (Bi_2_S_3_ + X-ray), which demonstrated the crucial role of 3BP on RT. To prove the synergistic effect (not only additive effect) of 3BP and Bi_2_S_3_-based RT, the CI was calculated. When the influence score (*f*_a_) = 0.5, the CI of 3BP and Bi_2_S_3_-based RT was 0.46, indicating that 3BP and Bi_2_S_3_-based RT had a significant synergistic effect after integrating Bi_2_S_3_-3BP + X-ray.

After the reduction of oxygen consumption by 3BP, more intracellular oxygen is available for RT. Intracellular ROS generation during radiotherapy was investigated using DCFH-DA as a fluorescent ROS probe. Cells treated with Bi_2_S_3_-3BP + X-ray showed a distinct green fluorescence after X-ray irradiation (Fig. 3b). In contrast, only slight green fluorescence was observed in cells treated with Bi_2_S_3_ + X-ray, indicating the contribution of 3BP for ROS generation and providing a solid basis for achieving RT enhancement under hypoxic conditions. The γ-H2AX focus in the nucleus is commonly used as a quantitative marker for DNA double-strand breaks. To demonstrate the level of DNA damage, cells after various treatments were stained with γ-H2AX for CLSM observation. A significant increase in γ-H2AX focus was observed after Bi_2_S_3_-3BP + X-ray pretreatment under both normoxic and hypoxia conditions (Fig. 3c-d). Furthermore, the proliferation of tumor cells under X-ray irradiation was assessed by cell colony formation assay (CFA). Tumor cells were incubated with different drugs for 4 h under normoxic and hypoxia conditions, then irradiated with X-ray. The formed cell colonies were counted after 7 days. Under normoxic conditions, Bi_2_S_3_ + X-ray group had significantly fewer colonies (284 clones) than the X-ray-only group (836 clones), indicating that Bi_2_S_3_ can effectively sensitize RT. In contrast, the number of colonies in the Bi_2_S_3_ + X-ray group (436 clones) was higher under hypoxia than in the normoxic group, suggesting that hypoxia affects the efficacy of RT. The number of cell colonies (normoxic: 159 clones, hypoxia: 106 clones) was the lowest in the Bi_2_S_3_-3BP + X-ray treated group under both normoxic and hypoxia conditions (Fig. 3e-f). The data consistently demonstrated the important role of these 3BP-containing nanospheres on RT.

### Pro-death atophagy induced by Bi_2_S_3_-3BP

**Fig. 4:**
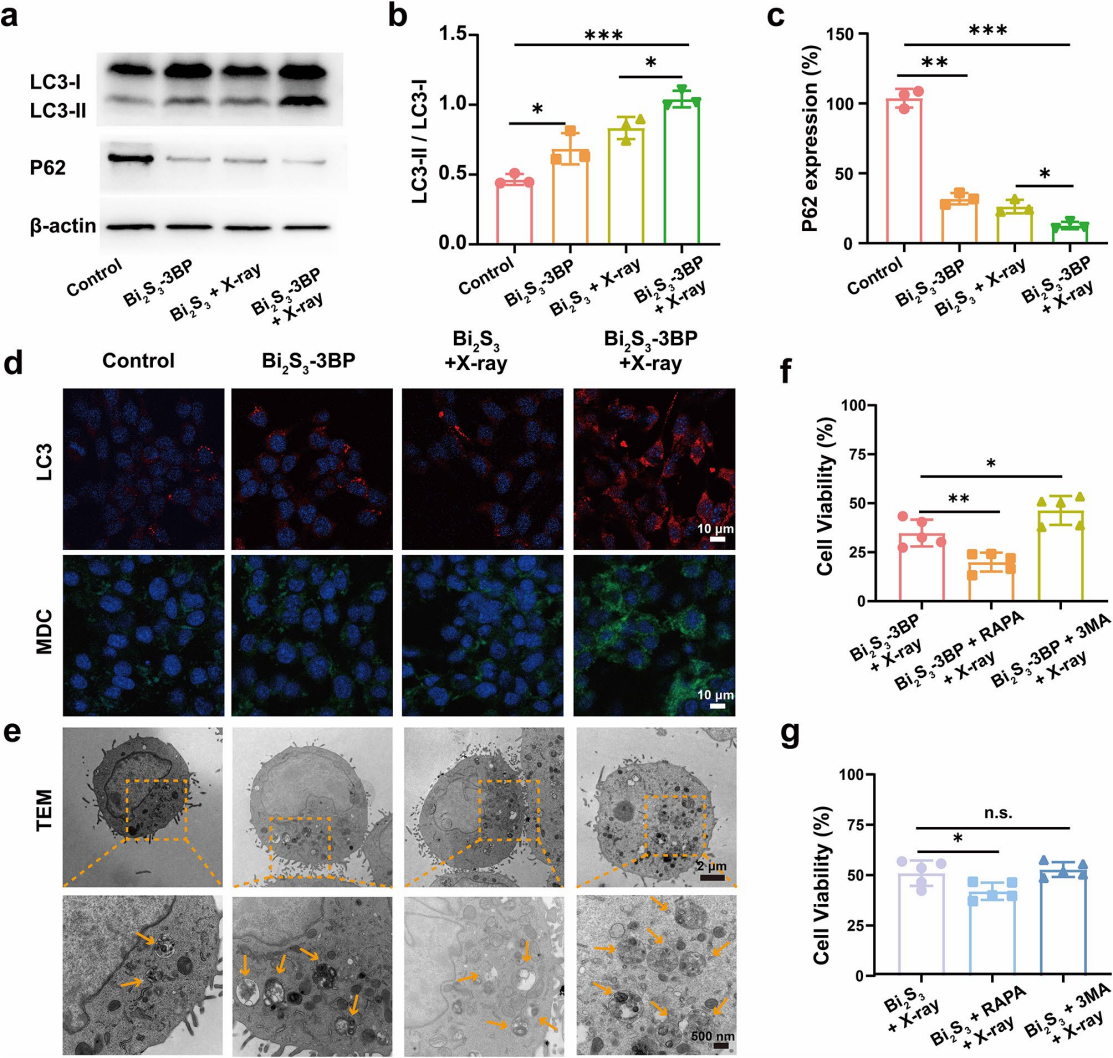
Pro-death autophagy of Bi_2_S_3_-3BP. **(a)** LC3-II, LC3-I, and P62 expression after different treatments as measured by western blot. **(b-c)** Quantitative analysis of relative LC3-II/LC3-1 and P62 expression. **(d)** Representative immunofluorescence images of LC3 punctate dots and MDC in different groups. **(e)** TEM images showing the formation of autophagosomes after different treatments. Zoomed-in TEM images in typical structures of autophagosomes are indicated with arrows. **(f-g)** Cell viability of 4T1 cells after being treated with Bi_2_S_3_ + X-ray or Bi_2_S_3_-3BP + X-ray in the presence of RAPA or 3-MA (n = 5). n.s.: no significance, *P < 0.05, **P < 0.01, ***P < 0.001

Indeed, autophagy can be activated under conditions of nutrient starvation as well as oxidative stress through highly regulated pathways associated with energy metabolism [38, 39]. Dysregulation of redox homeostasis, resulting from excessive production of ROS induced by the combined effect of 3BP and RT, can induced the onset of pro-death autophagy, and further effectively improve the therapeutic outcome [40, 41]. In order to obtain insight into the role of autophagy during RT, we investigated cellular autophagy levels following different treatments. The expression level of autophagy-related proteins (LC3 and P62) were evaluated by western blot assay. During autophagy, LC3-I is converted into LC3-II, while the autophagic substrate P62 is degraded. The LC3-II/LC3-I ratio in the combined treatment group (Bi_2_S_3_-3BP + X-ray group) was higher than that in the Bi_2_S_3_-3BP group and Bi_2_S_3_ + X-ray group, while the expression of P62 was reduced to 13.8% (Fig. 4a - c). Furthermore, the level of autophagy was investigated by visualizing and tracking LC3 puncta using immunofluorescence imaging (Fig. 4d). Compared with Bi_2_S_3_-3BP group and Bi_2_S_3_ + X-ray group, the red fluorescence was notably enhanced in Bi_2_S_3_-3BP + X-ray group. Acidic vesicular autophagosomes generated during autophagy could be investigated by MDC staining (Fig. 4f). The Bi_2_S_3_-3BP + X-ray group resulted in much stronger green fluorescence of acidic vesicular autophagosomes than that in Bi_2_S_3_-3BP group and Bi_2_S_3_ + X-ray group. These results indicated that the combination of Bi_2_S_3_-3BP and RT significantly increased the level of intracellular autophagy. The accumulation of autophagosomes was directly observed by TEM (Fig. 4e), while there were fewer autophagosomes in the control group. In contrast, multiple autophagosomes were clearly observed in the Bi_2_S_3_-3BP + X-ray group, suggesting the evident presence of autophagy.

To investigate whether over-activated autophagy plays a pro-death or pro-survival role during RT, the cytotoxicity of different treatments was examined after the addition of autophagy initiators (Rapa) and autophagy inhibitors (3-MA). Rapa or 3-MA alone showed almost no cytotoxicity (Figure S10). The CCK-8 assay results showed that cytotoxicity was slightly enhanced in the Bi_2_S_3_ + X-ray group after Rapa promoted autophagy (Fig. 4g). In contrast, after 3-MA inhibited autophagy, the cytotoxicity of Bi_2_S_3_ + X-ray was not significantly affected. However, when Bi_2_S_3_-3BP was combined with RT, 3-MA inhibition of autophagy enhanced the survival of 4T1 cells, while the cytotoxicity of Bi_2_S_3_-3BP + X-ray was significantly enhanced after Rapa promoted-autophagy (Fig. 4f). Taken together, the integration of 3BP into RT can promote the occurrence of pro-death autophagy in cells, which holds great promise in improving the therapeutic efficacy.

### PA and CT imaging

Effective accumulation of Bi_2_S_3_-3BP at the tumor site is essential for subsequent in vivo radiation therapy. Therefore, monitoring the biodistribution of Bi_2_S_3_-3BP by real-time imaging is necessary. Considering the excellent NIR optical absorption properties of Bi_2_S_3_-3BP, it could act as a PA contrast agent [42]. For PA imaging assessments, 715 nm was selected as the optimal wavelength for Bi_2_S_3_-3BP (Figure S11). The PA signal strength of Bi_2_S_3_-3BP increased linearly from 0.301 to 1.147 with increasing Bi_2_S_3_-3BP concentration (Figure S12). Further in vivo PA imaging was performed to detect the accumulation of Bi_2_S_3_-3BP in the tumor tissue. A gradual increase in PA signal was observed in the tumor region after intravenously administration, and peaked at 6 h after injection (Figure S13). The quantitative analysis result is also corresponding well with PA imaging (Figure S14).

Given the excellent X-ray attenuation properties and high biocompatibility of elemental bismuth [43], Bi_2_S_3_-3BP is also expected to be an ideal contrast agent for CT imaging. Therefore, we systematically investigated the in vitro and in vivo CT contrast imaging of Bi_2_S_3_-3BP. Similar to the PA results, the CT enhancement showed a good linear correlation with Bi_2_S_3_-3BP concentration (Figure S15). After intravenously administration of Bi_2_S_3_-3BP, a significant bright effect was observed in the tumor area (Figure S16), indicating the effective accumulation of Bi_2_S_3_-3BP in the tumor site.

### In vivo hypoxia relief, ROS generation and autophagy

**Fig. 5:**
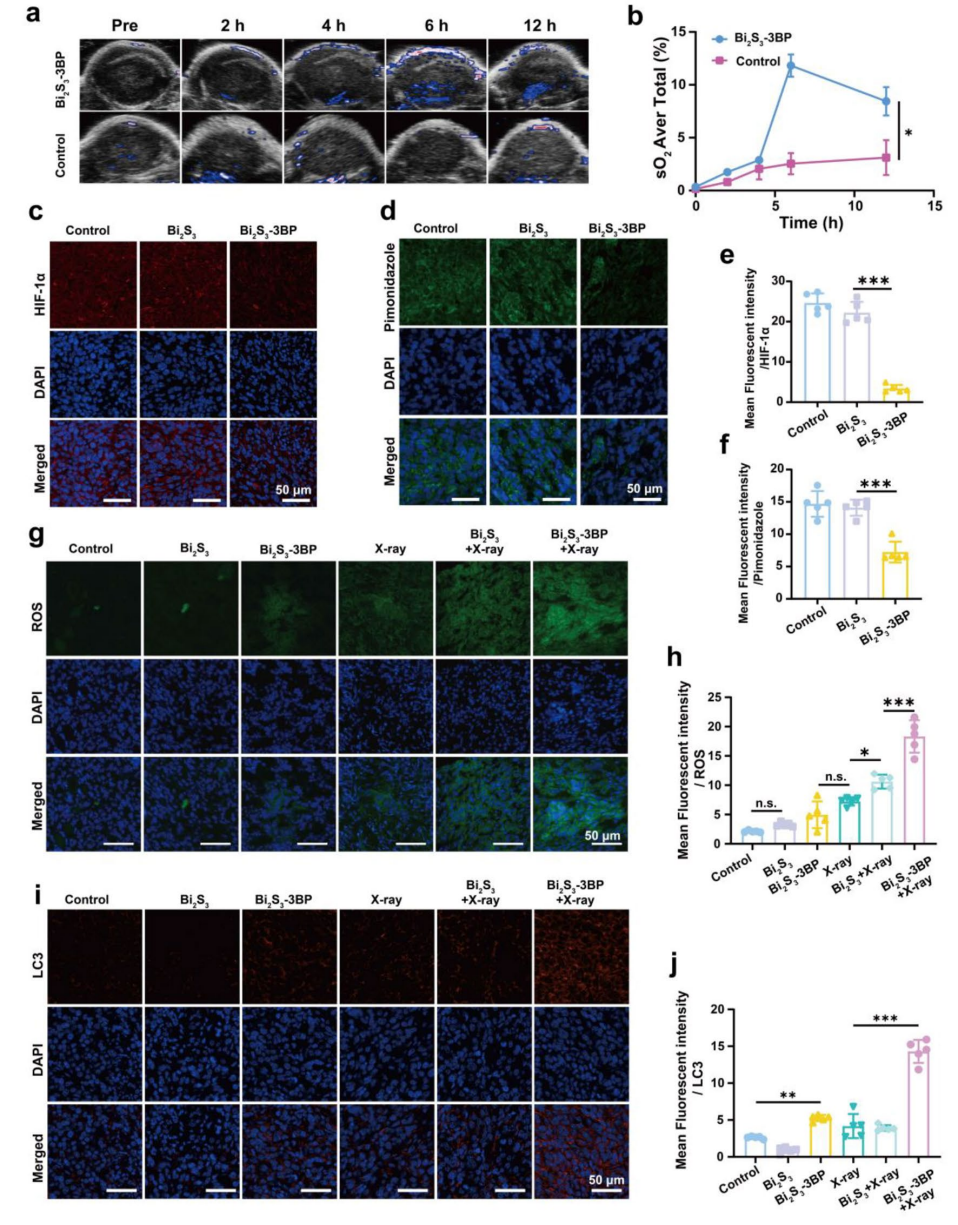
In vivo hypoxia relief, ROS generation and autophagy. **(a)** PA images of tumor sites in oxy-hemoglobin mode at different time points. **(b)** Quantification of oxyhemoglobin saturation at tumor sites (n = 3). **(c-d)** Immunochemical staining of HIF-1α and pimonidazole on tumor sections from 4T1 tumor-bearing mice after various treatments. **(e-f)** Mean fluorescence intensity semiquantitative analysis of HIF-1α and pimonidazole (n = 5). **(g-j)** Immunochemical staining of ROS and LC3 on tumor sections from 4T1 tumor-bearing mice, and the corresponding semiquantitative analysis (n = 5). n.s.: no significance, *P < 0.05, **P < 0.01, ***P < 0.001

In vitro assessments demonstrated that Bi_2_S_3_-3BP could effectively alleviate cellular hypoxia, and we expect to exhibit a similar effect in vivo. The ongoing changes of tumor hypoxia levels were monitored using oxyhemoglobin signals captured by the PA imaging system. As shown in Fig. 5a, the oxyhemoglobin signal gradually increased and peaked at 6 h after intravenously administration of Bi_2_S_3_-3BP. The result also showed a significantly higher signal than that of the control group. Quantitative analysis (Fig. 5b) showed that the oxyhemoglobin signal intensity of Bi_2_S_3_-3BP group increased from 0.3% to 11.8% at 6 h post-injection, while the control group (treated with saline) showed almost no change. HIF-1α immunofluorescence staining was also employed to confirm the ability of Bi_2_S_3_-3BP to relieve tumor hypoxia in vivo. Extensive red fluorescence was observed in both the saline and Bi_2_S_3_ groups, suggesting the intratumoral hypoxia. In contrast, the tumor area exhibited only a faint red fluorescence after intravenous injection of Bi_2_S_3_-3BP (Fig. 5c). Semi-quantitative analysis showed that the fluorescence intensity of tumor tissues after Bi_2_S_3_-3BP injection was reduced by 21% as compared to the control group (Fig. 5e). Furthermore, pimonidazole was used as the hypoxic probe to detect the hypoxia status, where hypoxic areas tend to exert green fluorescence. Corresponding to HIF-1α fluorescence, the group treated with Bi_2_S_3_-3BP exhibited much weaker green fluorescence than the groups treated with saline and Bi_2_S_3_ (Fig. 5d and f). These results well verified that 3BP could effectively relieve tumor inner hypoxia status, which is favorable for enhanced RT.

Next, ROS in vivo generation was measured. Mice bearing 4T1 tumors were randomly divided into six groups, including the control group (treated with saline), Bi_2_S_3_ only group, Bi_2_S_3_-3BP only group, X-ray only group, Bi_2_S_3_ + X-ray group, and Bi_2_S_3_-3BP + X-ray group. Much stronger green fluorescence of ROS was observed in Bi_2_S_3_-3BP + X-ray group as compared with other groups (Fig. 5g). Semi-quantitative analysis (Fig. 5h) showed that the fluorescence intensity of ROS in the Bi_2_S_3_-3BP + X-ray group was twice higher than that of Bi_2_S_3_ + X-ray group. This result indicated that Bi_2_S_3_-3BP + X-ray group could effectively alleviate tumor hypoxia and stimulate a substantial increase in ROS production, thereby amplifying the therapeutic efficacy of RT.

We further used immunofluorescence imaging of LC3 in tumor sections to assess the level of autophagy following various treatments in vivo. Notably, the Bi_2_S_3_-3BP + X-ray group showed significantly higher red fluorescence levels than other treatments (Fig. 5i). Semi-quantitative analysis (Fig. 5j) showed that the fluorescence intensity of LC3 in the Bi_2_S_3_-3BP + X-ray group was 3.6 times higher than that of Bi_2_S_3_ + X-ray group. This finding suggested that the Bi_2_S_3_-3BP + X-ray group induced a higher level of autophagy. Overall, these results indicate that 3BP can alleviate hypoxia and enhance the autophagy occurrence in tumor cells during RT, thus maximizing the therapeutic effect.

**Fig. 6:**
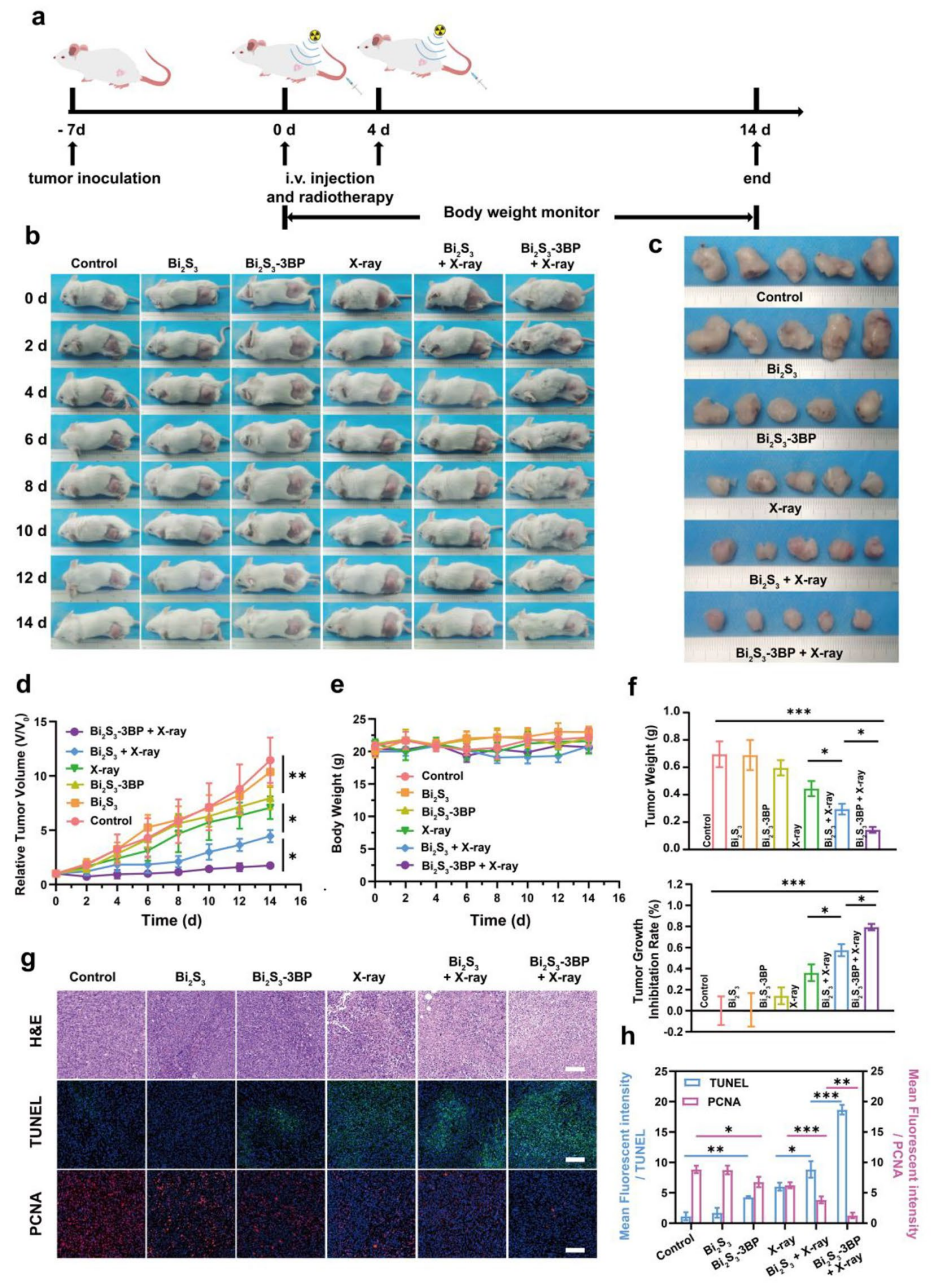
Anti-tumor efficacy in vivo. **(a)** Schematic illustration of the Bi_2_S_3_-3BP-based RT process. **(b)** Representative photographs of 4T1 tumor-bearing mice of six groups during the 14 d period. **(c)** Photographs of tumors dissected from mice of six groups after various treatments. **(d)** Tumor growth curves of six groups after various treatments. (n = 5). **(e)** Body-weight curves of six groups during the observation period. (n = 5). **(f)** Weight of tumors at 14 d post various treatments and tumor growth inhibition rate (n = 5). **(g)** H&E, TUNEL and PCNA staining of tumors from 4T1 tumor-bearing mice after various treatments. **(h)** Mean fluorescence intensity semiquantitative analysis of PCNA and TUNEL (n = 5). *P < 0.05, **P < 0.01, ***P < 0.001

### Anti-tumor efficacy in vivo

Encouraged by the in vitro therapeutic efficacy of Bi_2_S_3_-3BP-mediaed RT, as well as the in vivo hypoxia relief, ROS generation, and autophagy results, the anti-tumor efficacy in vivo was investigated next. Tumor-bearing mice were randomly divided into six groups, including the control group (treated with saline), Bi_2_S_3_ only group, Bi_2_S_3_-3BP only group, X-ray only group, Bi_2_S_3_ + X-ray group, and Bi_2_S_3_-3BP + X-ray group. Nanospheres were intravenously injection into mice on days 0 and 4, with X-ray irradiation administrated to the tumor site at 6 h after the injection (Fig. 6a). Tumor volumes were monitored after different treatments (Fig. 6b and c), and normalized by relative tumor volume (V/V_0_) (Fig. 6d).

It was found that the Bi_2_S_3_ group did not show significant tumor growth inhibition (Fig. 6f). Conventional RT without any sensibilization showed a slight tumor suppressive effect with a tumor growth inhibition rate of 36%. However, when Bi_2_S_3_ as an ionizing radiation sensitizer was introduced, the therapeutic efficacy of RT in the Bi_2_S_3_ + X-ray group was significantly enhanced, achieving a tumor growth inhibition rate of 57%. In contrast, tumors treated with Bi_2_S_3_-3BP + X-ray group were significantly reduced, with a tumor growth inhibition rate reaching 79.3%. This could be ascribed to ionizing radiation enhancement, oxygen consumption reduction, and pro-death autophagy activation, which sensitized the cells to RT. Consistently, in vitro tumor visualization (Fig. 6c) and weight (Fig. 6f) followed a similar trend with no significant body weight loss during the 14-day observation period (Fig. 6e).

In addition, H&E, TUNEL, and PCNA staining were performed to test the therapeutic efficacy at a pathological level (Fig. 6g). HE staining of tumor sections showed that the Bi_2_S_3_-3BP + X-ray group had significantly more deformed cell nuclei (nuclear consolidation, nuclear fragmentation, and nuclear lysis) than tumor sections from other groups, indicating severe necrosis of cancer cells. The level of apoptosis observed with TUNEL (green fluorescence) was consistent with that of H&E staining. TUNEL staining showed that Bi_2_S_3_-3BP + X-ray group induced more apoptotic cells than the Bi_2_S_3_ + X-ray and X-ray only groups. In contrast, the trend was reversed when examining PCNA staining (red fluorescence), which reflects the level of cell proliferation.

Finally, semi-quantitative analysis of apoptotic and proliferative regions (Fig. 6h) showed that the fluorescence intensity of PCNA in the Bi_2_S_3_ + X-ray group was 3-fold higher than that of Bi_2_S_3_-3BP+ X-ray group, while the fluorescence intensity of TUNEL for the Bi_2_S_3_ + X-ray was only one-third of Bi_2_S_3_-3BP + X-ray group. These results suggest that enhanced RT efficiency was achieved in 4T1 breast cancer tumors when incorporating Bi_2_S_3_-3BP nanospheres, as compared with the conventional RT without any sensitization method.

## Conclusion

In this study, we successfully designed Bi_2_S_3_-3BP nanospheres to effectively sensitize the impact of RT by enhancing ionizing radiation, reducing oxygen consumption and activating pro-death autophagy in tumor cells. These nanospheres are enriched in tumor tissues through the EPR effect, as monitored by CT/PA imaging. The Bi element acted as a radiation therapy sensitizer, effectively absorbing ionizing radiation during RT treatment. Meanwhile, 3BP effectively alleviated the hypoxia status of tumor tissues by inhibiting the tumor glycolytic metabolic pathway and mitochondrial OXPHOS, further aggravating the DNA damage during RT. Additionally, the ROS generation induced by the combined effect of RT and 3BP increased the ROS level. The cellular autophagy induced by the overproduction of intracellular ROS and the starvation effect of 3BP exhibited a pro-death effect, further optimized the final outcome of RT. We compared Bi_2_S_3_ + X-ray with Bi_2_S_3_-3BP + X-ray under the same experimental conditions, and the cell survival rate of Bi_2_S_3_-3BP + X-ray was only a quarter of Bi_2_S_3_ + X-ray. Although in vitro effects of different metal nanoparticles in radiation therapy have been observed in previous studies, few studies have combined metal nanomaterials with autophagy. Our study achieved desirable anti-tumor effects at both in vitro and in vivo. It may provide a new idea for the study of nanoradiosensitizers and offer a strategy for a precision radiation oncology approach for clinical tumor radiosensitization.

## Electronic supplementary material

Below is the link to the electronic supplementary material.

**Additional file: Fig S1-S2** TEM images of Bi_2_O_3_ nanoparticles and Bi_2_S_3_ nanospheres. **Fig. S3** The porosity of Bi_2_S_3_ nanospheres. **Fig. S4** Stability of Bi_2_S_3_ nanospheres with or without PEG modification in PBS. **Fig. S5** The Tyndall effect of Bi_2_S_3_ nanospheres in PBS. **Fig. S6** (a) UV-vis absorbance spectra of 3BP at various concentrations. (b) The relative absorbance of 3BP at 282 nm against concentration. **Fig S7.** Cumulative release of 3BP from Bi_2_S_3_-3BP. **Fig. S8** HK-II viability of 4T1 cells. **Fig. S9** Mean Fluorescence intensity quantitative analysis of hypoxia red fluorescence. **Fig. S10** Cell viability of 4T1 cells after treat with Rapa and 3-MA. **Fig. S11** The recorded PA intensity of Bi_2_S_3_-3BP at λ = 700–950 nm. **Fig. S12** In vitro PA contrast images and PA values of Bi_2_S_3_-3BP at different concentrations. **Fig. S13** In vivo PA images of tumors in tumor-bearing mice after intravenously injection of Bi_2_S_3_-3BP at different time points. **Fig. S14** Changes of PA-signal intensities within tumor regions at corresponding time points. **Fig. S15** In vitro CT contrast images and HU values of Bi_2_S_3_-3BP at different concentrations. **Fig. S16** In vivo CT images of tumor-bearing mice after intravenously injection of Bi_2_S_3_-3BP at different time points

## Data Availability

All data used to obtain the present results are available within the paper and the Supporting Information.
