# Supplementary material for: 3-Bromopyruvate-loaded bismuth sulfide nanospheres improve cancer treatment by synergizing radiotherapy with modulation of tumor metabolism
Source: J Nanobiotechnology. 2023 Jul 5;21:209. doi: 10.1186/s12951-023-01970-8 (PMC10321018; doi:10.1186/s12951-023-01970-8)
Supplement: Supplementary file 1 — Additional file: Fig S1-S2 TEM images of Bi2O3 nanoparticles and Bi2S3 nanospheres. Fig. S3 The porosity of Bi2S3 nanospheres. Fig. S4 Stability of Bi2S3 nanospheres with or without PEG modification in PBS. Fig. S5 The Tyndall effect of Bi2S3 nanospheres in PBS. Fig. S6 (a) UV-vis absorbance spectra of 3BP at various concentrations. (b) The relative absorbance of 3BP at 282 nm against concentration. Fig S7. Cumulative release of 3BP from Bi2S3-3BP. Fig. S8 HK-II viability of 4T1 cells. Fig. S9 Mean Fluorescence intensity quantitative analysis of hypoxia red fluorescence. Fig. S10 Cell viability of 4T1 cells after treat with Rapa and 3-MA. Fig. S11 The recorded PA intensity of Bi2S3-3BP at λ = 700–950 nm. Fig. S12 In vitro PA contrast images and PA values of Bi2S3-3BP at different concentrations. Fig. S13 In vivo PA images of tumors in tumor-bearing mice after intravenously injection of Bi2S3-3BP at different time points. Fig. S14 Changes of PA-signal intensities within tumor regions at corresponding time points. Fig. S15 In vitro CT contrast images and HU values of Bi2S3-3BP at different concentrations. Fig. S16 In vivo CT images of tumor-bearing mice after intravenously injection of Bi2S3-3BP at different time points [file 12951_2023_1970_MOESM1_ESM.docx]

***Supplementary Information***

**3-Bromopyruvate-loaded Bismuth Sulfide Nanospheres Improve Cancer Treatment by Synergizing Radiotherapy with Modulation of Tumor Metabolism**

Yiman He^1^, Huawan Chen^2^, Wenbo Li^3^, Lu Xu^3^, Huan Yao^1^, Yang Cao^4^, Zhigang Wang^4^, Liang Zhang^1^, Dong Wang^1^**^*^**, Di Zhou^5^**^*^**

^1^Department of Ultrasound, the First Affiliated Hospital of Chongqing Medical University, Chongqing 400042, P.R. China

^2^Department of Oncology, the First Affiliated Hospital of Chongqing Medical University, Chongqing 400042, P.R. China.

^3^Department of Nuclear Medicine, the First Affiliated Hospital of Chongqing Medical University, Chongqing 400042, P.R. China.

^4^Chongqing Key Laboratory of Ultrasound Molecular Imaging, Institute of Ultrasound Imaging, The Second Affiliated Hospital, Chongqing Medical University, Chongqing 400010, P.R. China.

^5^Department of Radiology, the First Affiliated Hospital of Chongqing Medical University, Chongqing 400042, P.R. China.

*Correspondence: [zhoudi@cqmu.edu.cn](mailto:zhoudi@cqmu.edu.cn); wangdong4429@cqmu.edu.cn

**Additional Material and Methods**

**Measurement of HK-Ⅱ, GAPDH**

4T1 cells were seeded in a 6 cm cell culture dish at 4 × 10^5^ cells per dish for 24 h. Then PBS, Bi_2_S_3_ (Bi: 60 μg mL^-1^), Bi_2_S_3_-3BP (Bi: 60 μg mL^-1^, 3BP: 10 μg mL^-1^), 3BP only (10 μg mL^-1^), 2DG (500 μg mL^-1^), or rotenone (10 μg mL^-1^) were added and co-incubated for another 6 h. Subsequently, cells were lysed to extract protein. Then, the proteins of each group were separated by 10% sodium dodecyl sulfate (SDS) polyacrylamide gel electrophoresis (SDS-PAGE) and transferred onto the polyvinylidene fluoride (PVDF) membrane (EMD Millipore, Billerica, MA). The membranes were blocked with the blocking solution (Beyotime) for 15 min at room temperature. Then anti-glyceraldehyde-3-phosphate dehydrogenase (GAPDH) (Rabbit monoclonal antibody, 1:10000, Abcam) and HK-Ⅱ (Rabbit monoclonal antibody, 1:1000, Abcam) were added and placed at 4℃ for 12 to 14 h. Goat anti-rabbit IgG secondary antibody (1:4000, Biosharp, Guangzhou, China) was added, and incubated at room temperature for 1 h. Finally, the membranes were visualized using ECL (Bio-Rad, Hercules, CA). The intensity of each Western blot (WB) band was analyzed using the ImageJ and GraphPad Prism 8.0 software packages.

**Detection of cellular autophagy**

Expression of autophagy-related proteins P62 and LC3 was detected by WB assay. 4T1 cells were seeded in a 6 cm cell culture dish at 4 × 10^5^ cells per dish for 24 h. Then cells were treated with Control (untreated), Bi_2_S_3_-3BP (Bi: 60 μg mL^-1^, 3BP: 10 μg mL^-1^), Bi_2_S_3_ (Bi: 60 μg mL^-1^) + X-ray, and Bi_2_S_3_-3BP (Bi: 60 μg mL^-1^, 3BP: 10 μg mL^-1^) + X-ray, respectively. After incubated for another 6 h, cells in the X-ray group were subjected to radiotherapy. After another 1 h incubation, then cells were lysed to extract protein, the proteins of different groups were then separated by 12.5% sodium dodecyl sulfate (SDS) polyacrylamide gel electrophoresis (SDS-PAGE) and transferred onto the polyvinylidene fluoride (PVDF) membrane (EMD Millipore, Billerica, MA). The membranes were blocked with the blocking solution (Beyotime) for 15 min at room temperature. Then LC3A/B (Rabbit monoclonal antibody, 1:1000, Cell Signaling Technology, CST) or anti-P62 antibody (Rabbit monoclonal antibody, 1:2000, Abcam) was added, and placed at 4℃ for 12 to 14 h. Goat anti-rabbit IgG secondary antibody (1:4000, Biosharp, Guangzhou, China) was added, and incubated at room temperature for 1 h. Finally, the membranes were visualized using ECL (Bio-Rad, Hercules, CA). The intensity of each WB band was analyzed using the ImageJ and GraphPad Prism 8.0 software packages.

For immunofluorescence staining of LC3 dots, 4T1 cells were seeded in CLSM dishes for 4 groups: Control (untreated), Bi_2_S_3_-3BP (Bi: 60 μg mL^-1^, 3BP: 10 μg mL^-1^), Bi_2_S_3_ (Bi: 60 μg mL^-1^) + X-ray (6 Gy), and Bi_2_S_3_-3BP (Bi: 60 μg mL^-1^, 3BP: 10 μg mL^-1^) + X-ray. Cells were co-cultured with media containing Bi_2_S_3_ or Bi_2_S_3_-3BP for 6 h, and exposed to X-ray irradiation. After washing with PBS, the cells were fixed with 4% paraformaldehyde, permeabilized with PBS containing 0.1% (v/v) Triton X-100, and incubated with 5% bovine serum albumin. After that, the cells were incubated with LC3A/B (Rabbit monoclonal antibody, 1:1000, Cell Signaling Technology, CST) at 4 °C overnight, followed by incubating with Rhodamine (TRITC) – Conjugated Goat anti-rabbit IgG secondary antibody (1:100, ZSGB-BIO, Beijing, China) at 37 °C for 1 h. The cells were then dyed with DAPI for 15 min. The LC3 dots were monitored by a laser-scanning confocal microscope.

For the detection of cytotoxicity of autophagy inhibitor/promoter, 4T1 cells were seeded in a 96-well plate and treated as follows: Control (untreated), Rapa (3.5 μg mL^-1^), 3-MA (6 μg mL^-1^), respectively. After 24 h incubation, CCK-8 kit were used to detect the cell viabilities.

**PA and CT imaging of Bi_2_S_3_-3BP**

PA performance of Bi_2_S_3_-3BP was evaluated using the Vevo LAZR photoacoustic imaging system (Visual Sonic Inc., Toronto, Canada). PA imaging was performed on Bi_2_S_3_-3BP suspensions (10, 20, 40, 60, 80 and 100 μg mL^-1^, respectively), and the corresponding PA signal intensity was recorded. For *in vivo* PA imaging, 4T1 tumor-bearing mice (n = 3) were intravenously injected with Bi_2_S_3_-3BP suspension (Bi: 5 mg mL^-1^, 3BP: 0.8 mg mL^-1^, in 200 µL saline). Then, the PA images were collected at different time points (pre-injection, 2, 4, 6, and 12 h) and the average PA signal intensity of the tumor regions was measured.

CT imaging experiments were conducted on a clinical CT imaging system. Bi_2_S_3_-3BP suspension at different concentrations (0.3125, 0.625, 1.25, 2.5 and 5 mg mL^-1^, respectively) were placed in 5 mL eppendorf tubes for *in vitro* CT imaging. The imaging parameters were set to: 120 mA, 80 kV. The CT signal intensity within the region of interest was measured. For *in vivo* CT imaging, 4T1 tumor-bearing mice (n = 3) were intravenously injected with Bi_2_S_3_-3BP suspension (Bi: 5 mg mL^-1^,3BP: 0.8 mg mL^-1^, in 200 µL saline). CT images were then acquired at different time points (pre-injection and 6 h).


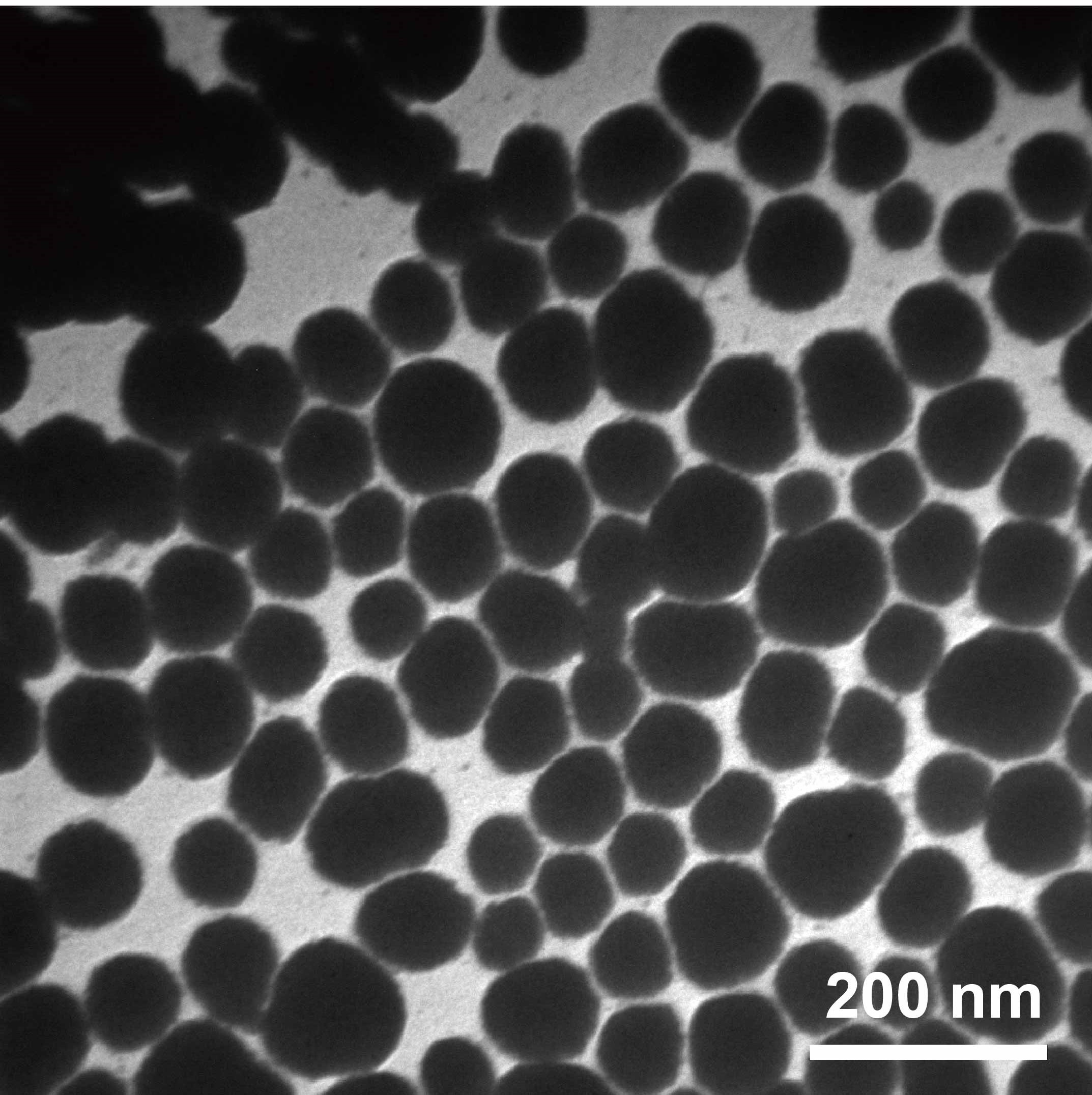


**Fig. S1.** TEM images of Bi_2_O_3_ nanoparticles.


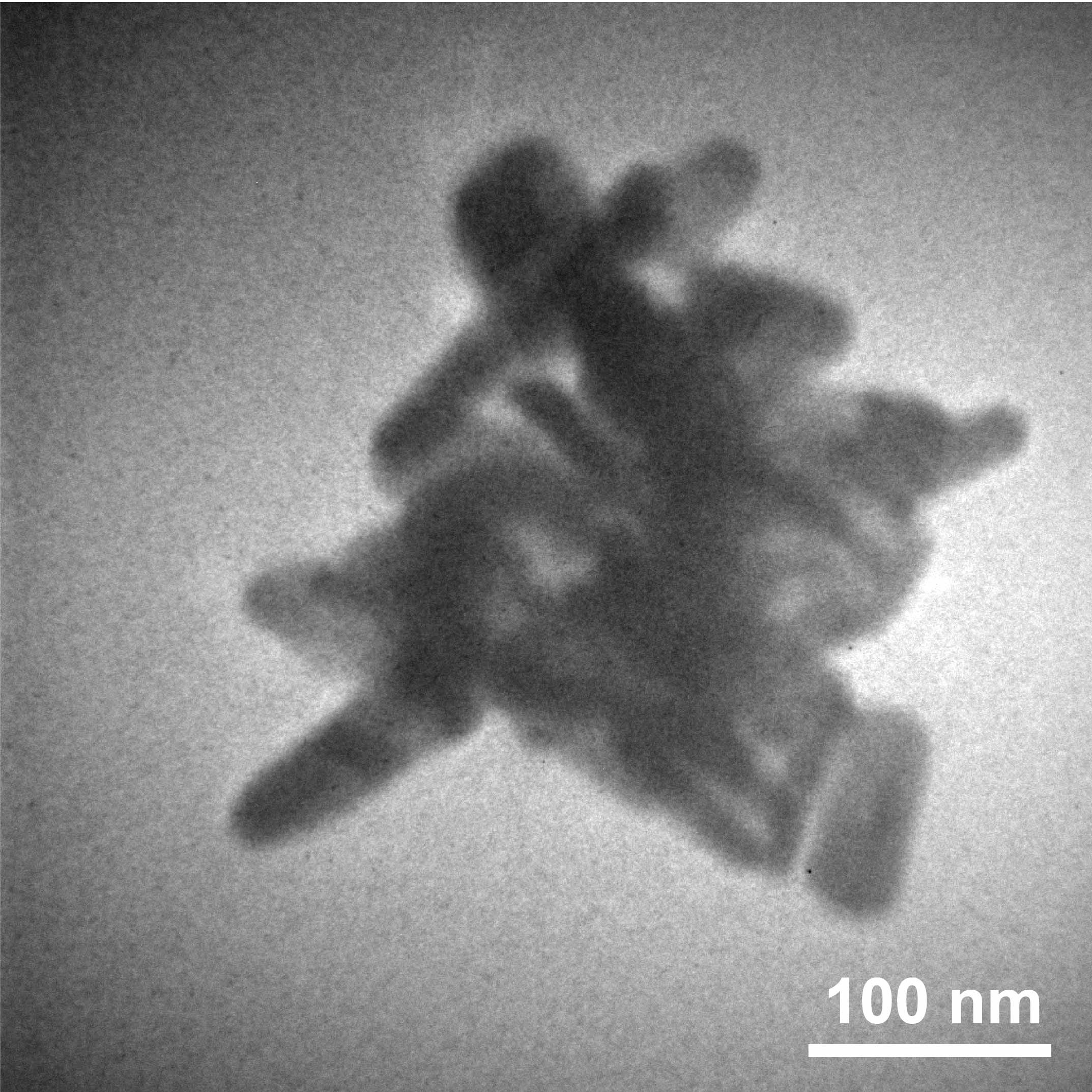


**Fig. S2.** TEM images of Bi_2_S_3_ nanospheres.


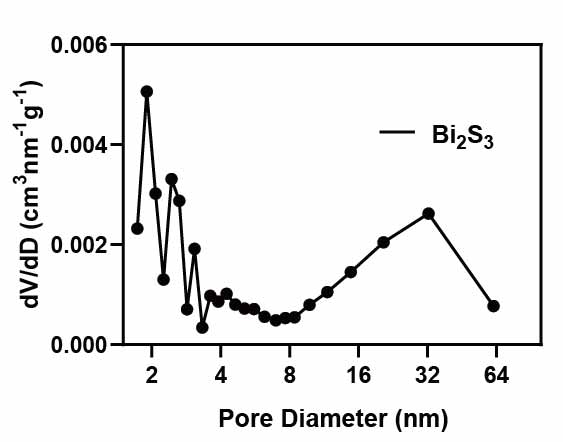


**Fig. S3.** The porosity of Bi_2_S_3_ nanospheres.


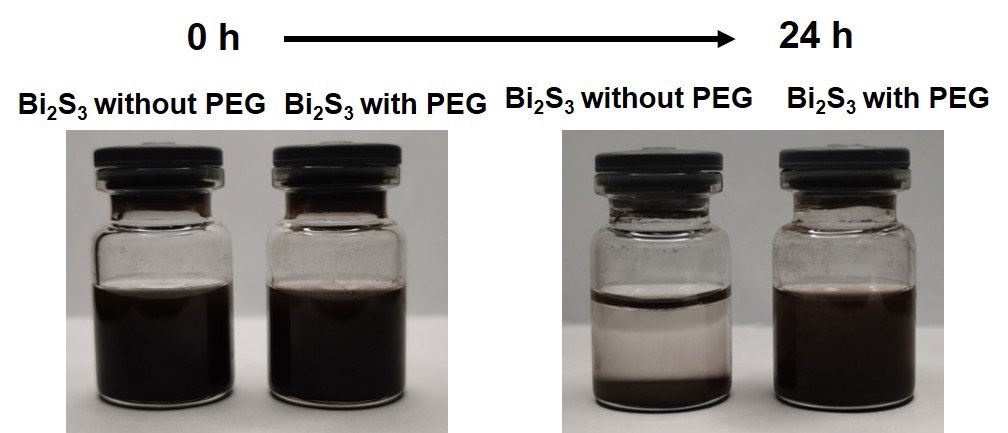


**Fig. S4.** The stability of Bi_2_S_3_ nanospheres with or without PEG modification in PBS.


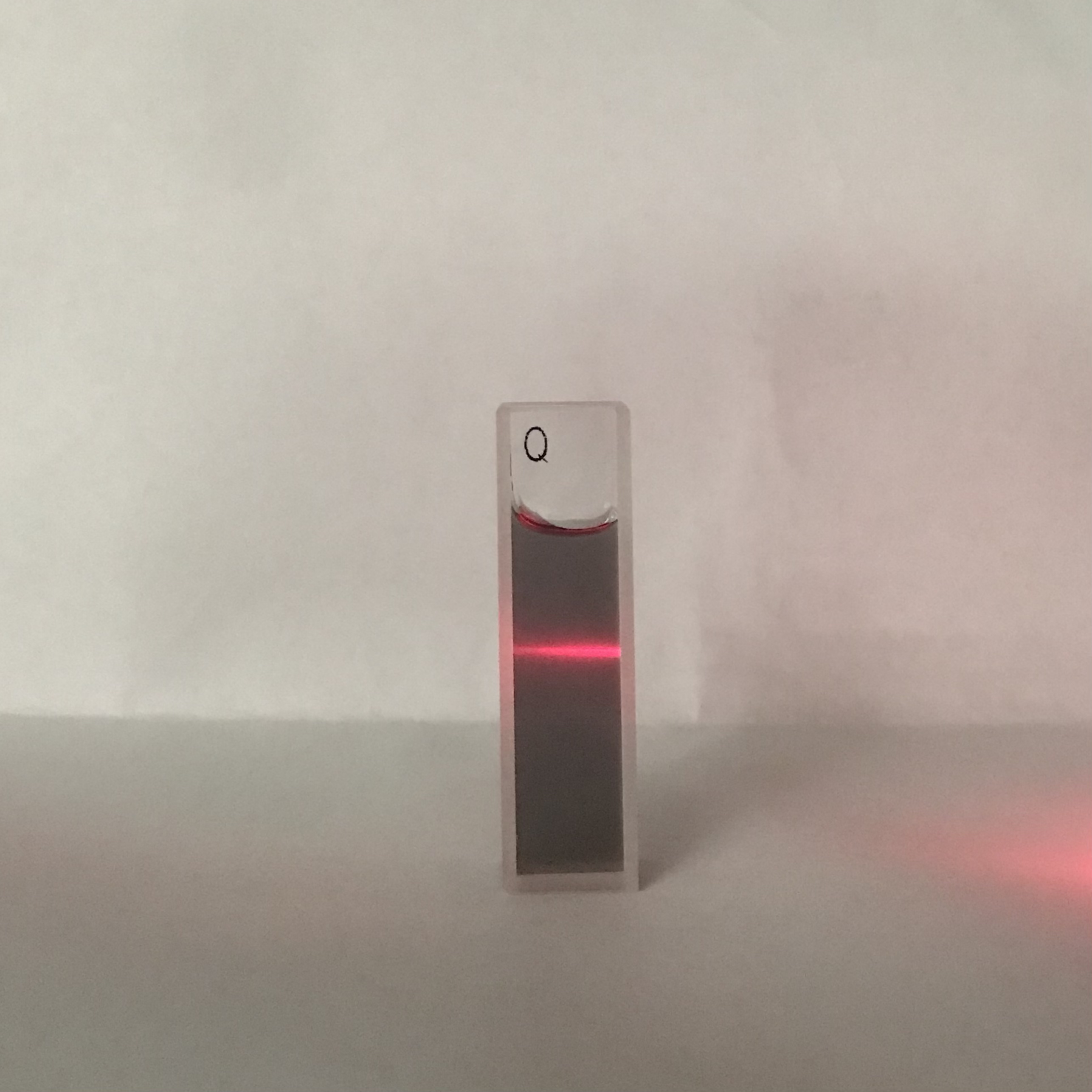


**Fig. S5.** The Tyndall effect of Bi_2_S_3_ nanospheres in PBS.


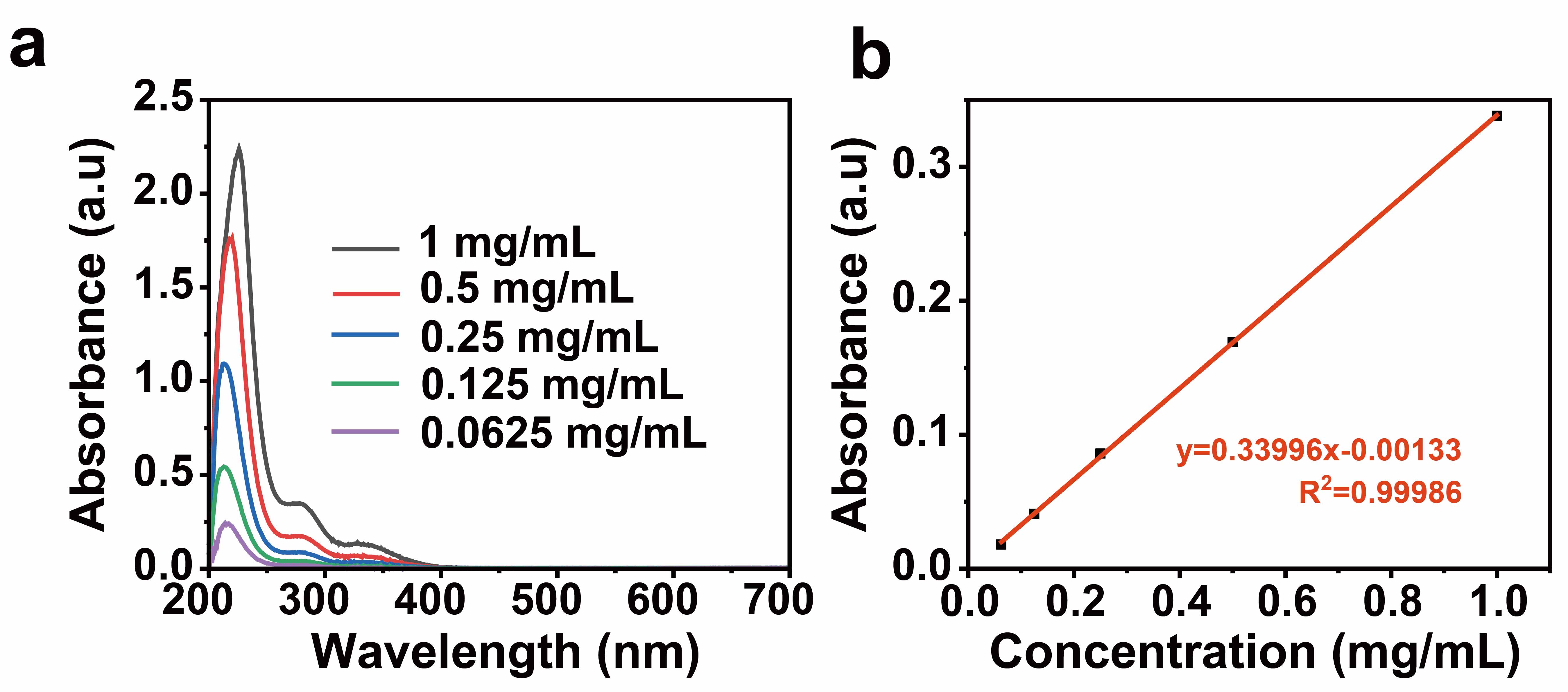


**Fig. S6.** (a) UV-vis absorbance spectra of 3BP at various concentrations. (b) The relative absorbance of 3BP at 282 nm against concentration.


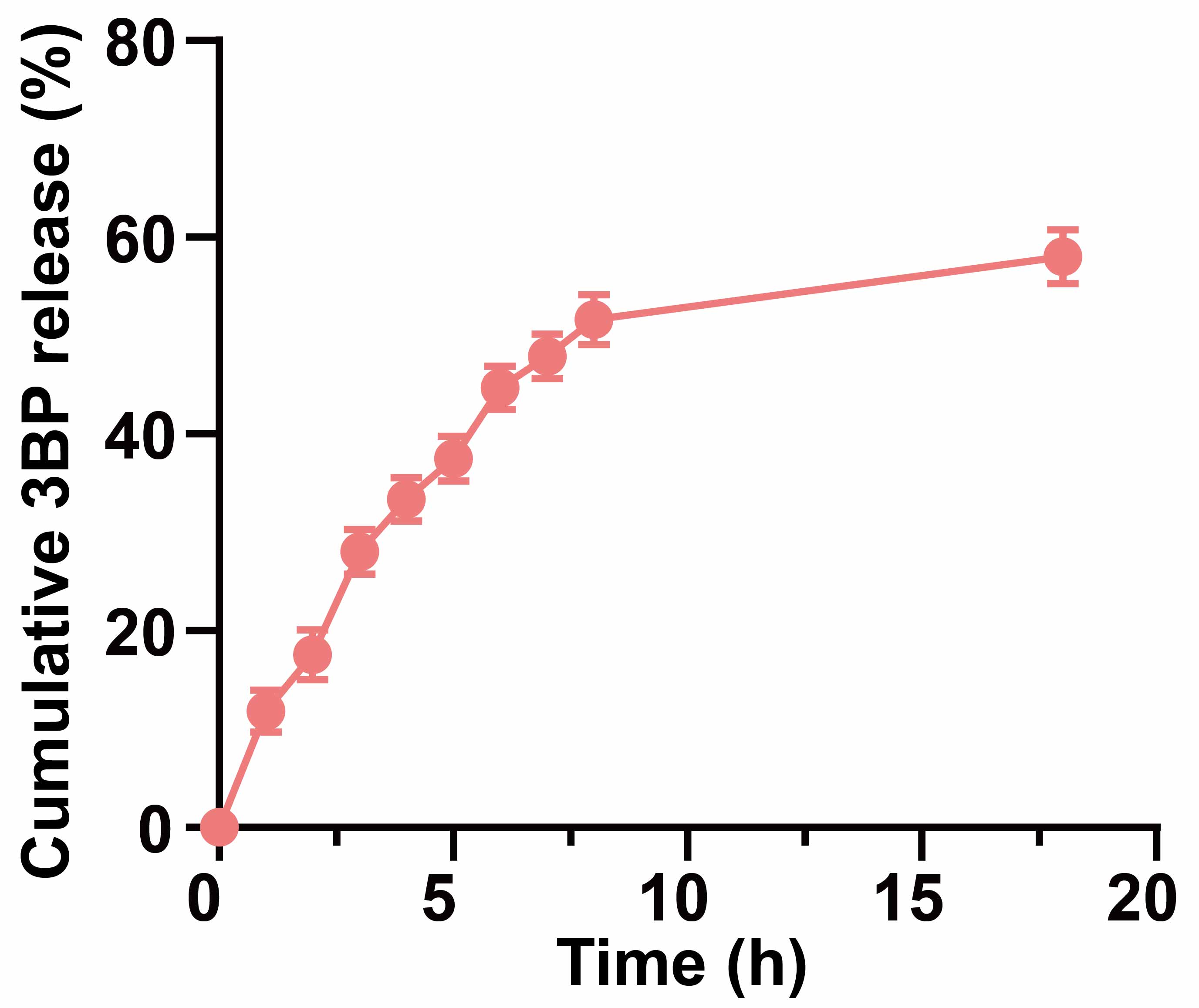


**Fig. S7.** Cumulative release of 3BP from Bi_2_S_3_-3BP (n = 3).


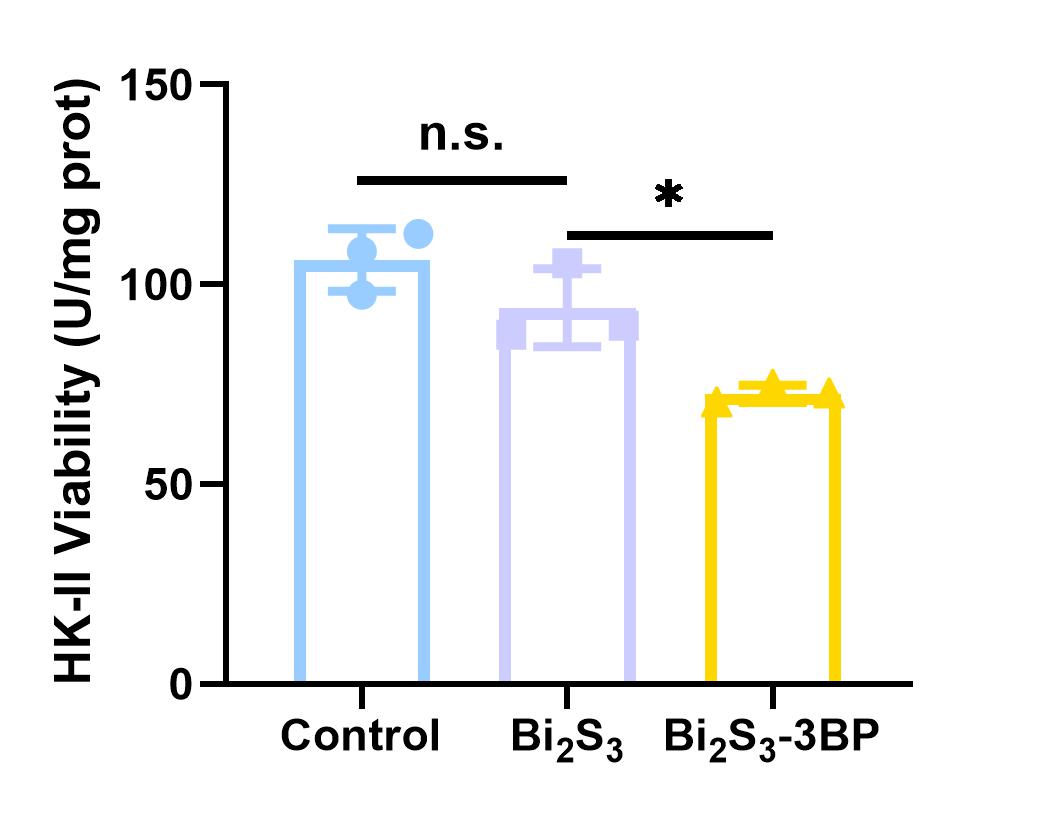


**Fig. S8.** HK-II viability of 4T1 cells (n = 3).


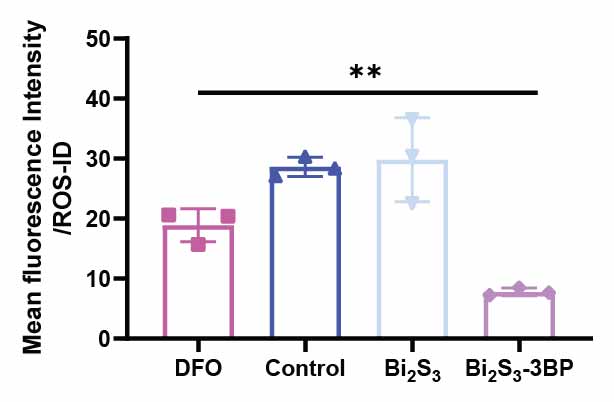


**Fig. S9.** Mean Fluorescence intensity quantitative analysis of hypoxia red fluorescence. (n = 3, **p < 0.01)


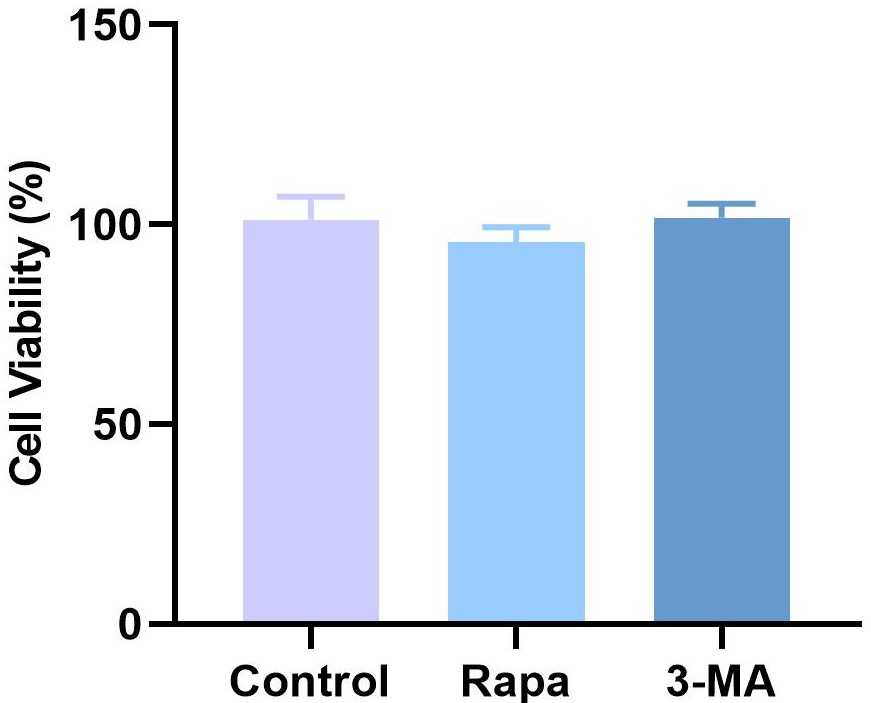


**Fig. S10.** Cell viability of 4T1 cells after treatment with Rapa and 3-MA. (n = 3)


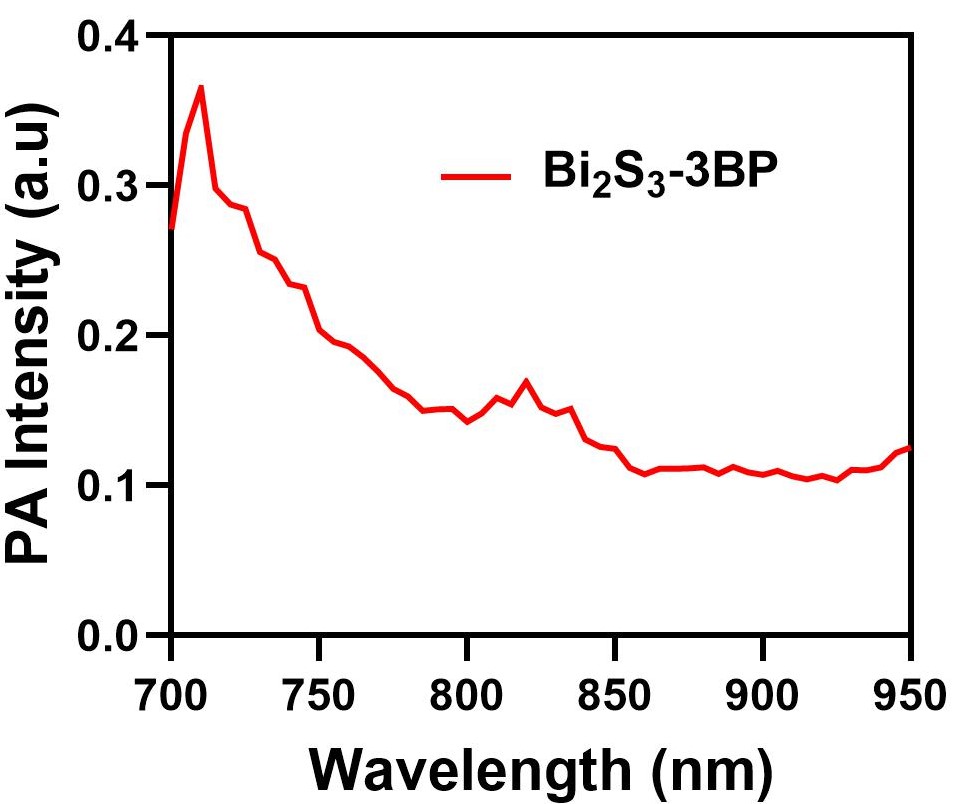


**Fig. S11**. The recorded PA intensity of Bi_2_S_3_-3BP at λ = 700 – 950 nm.


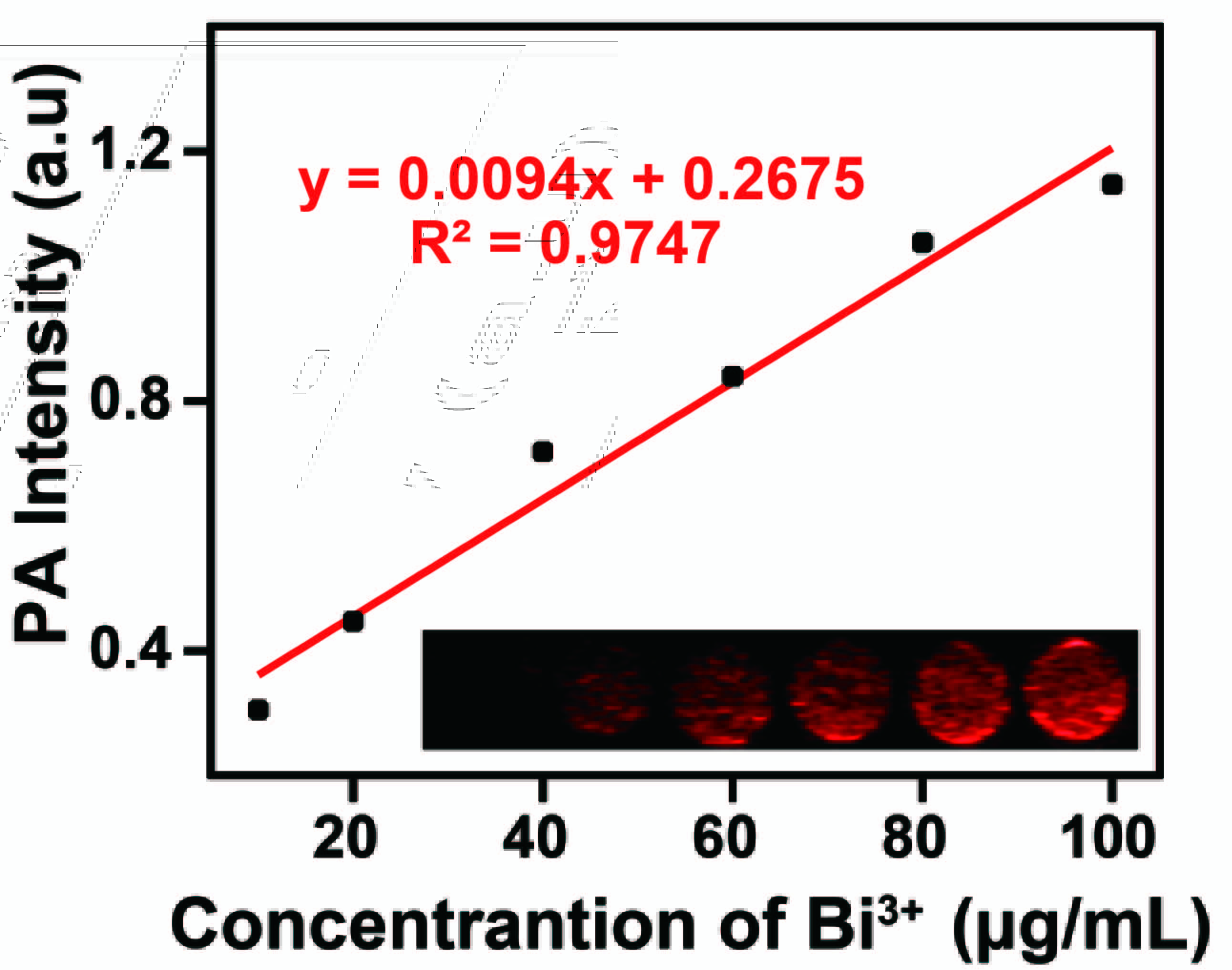


**Fig. S12.** *In vitro* PA contrast images and PA values of Bi_2_S_3_-3BP at different concentrations.


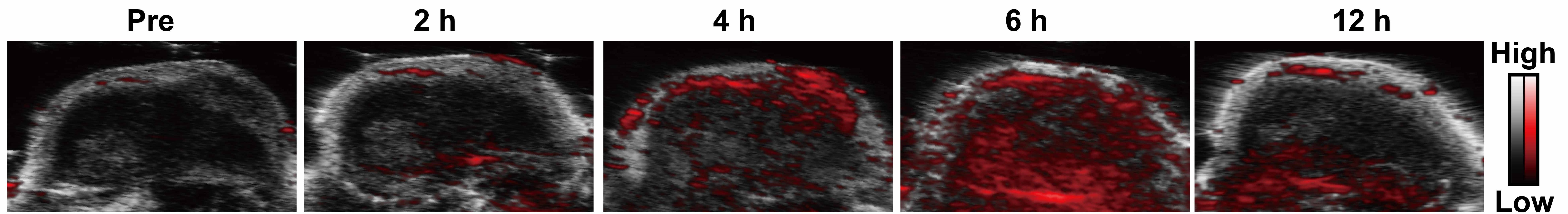


**Fig. S13.** *In vivo* PA images of tumors in tumor-bearing mice after intravenously injection of Bi_2_S_3_-3BP at different time points.


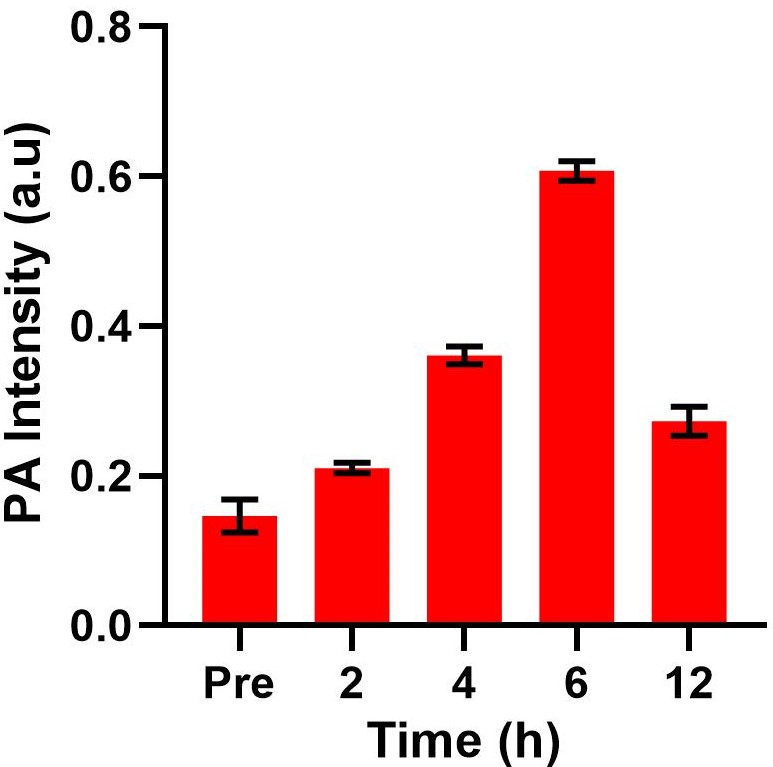


**Fig. S14.** Changes of PA-signal intensities within tumor regions at corresponding time points.


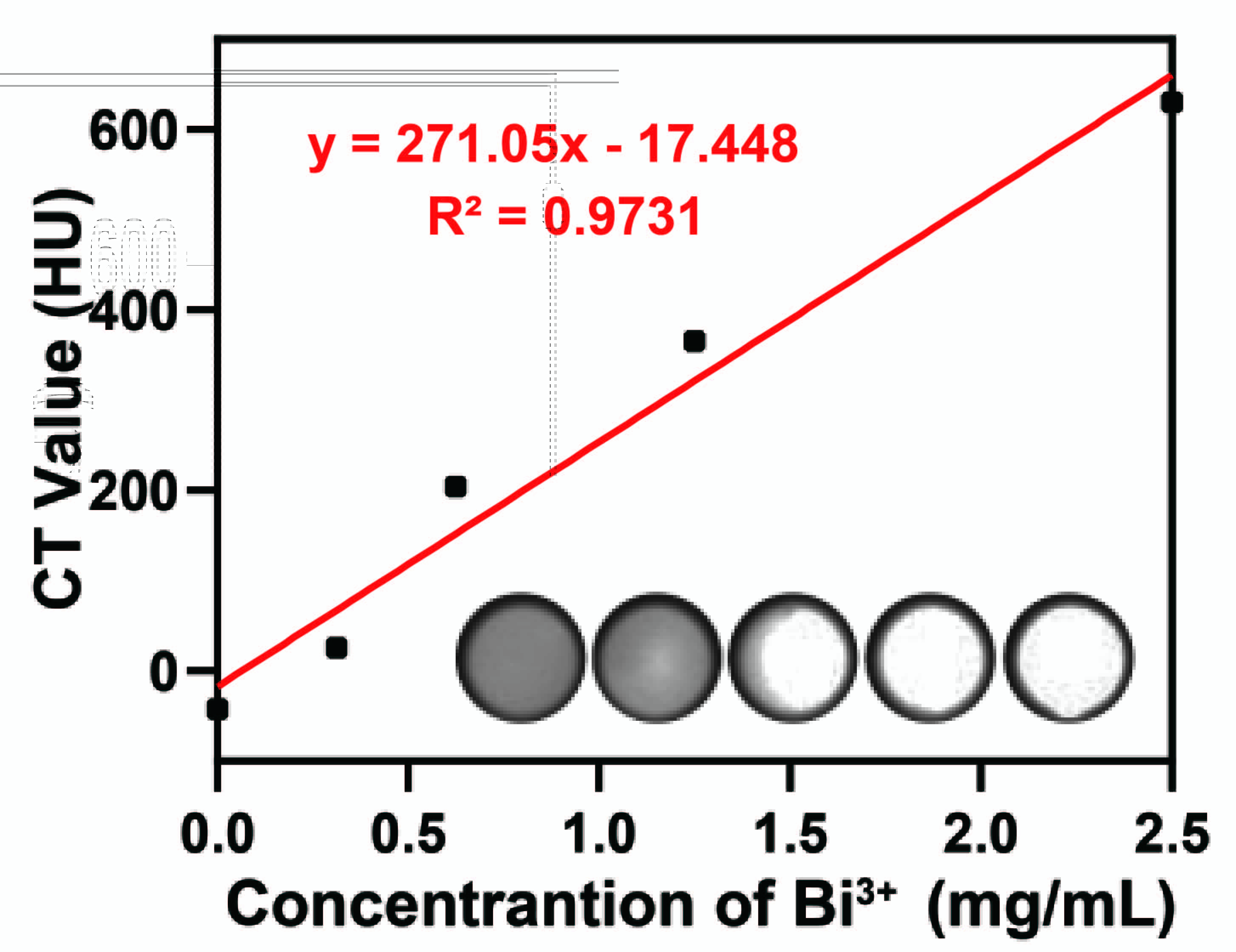


**Fig. S15.** *In vitro* CT contrast images and HU values of Bi_2_S_3_-3BP at different concentrations.


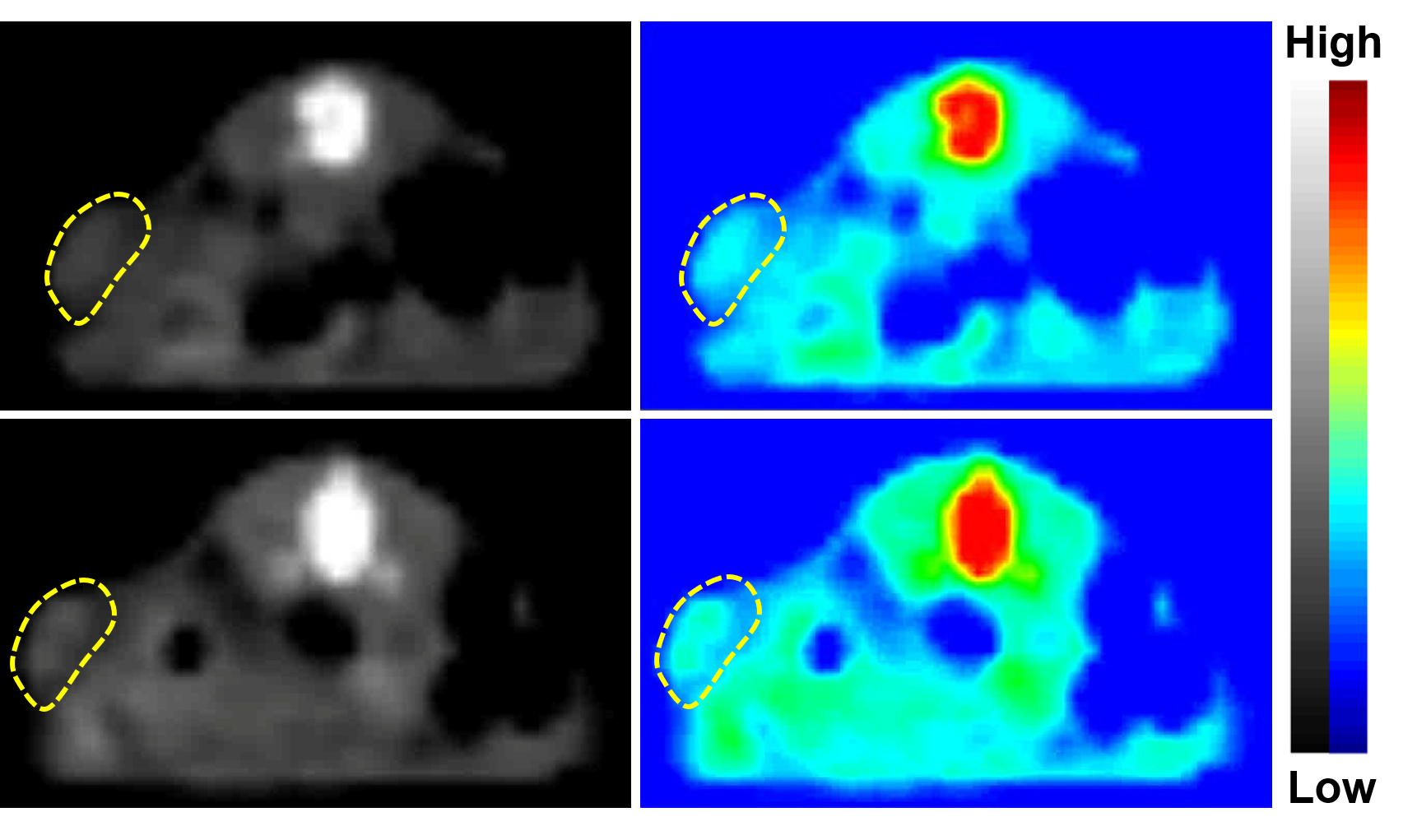


**Fig. S16.** *In vivo* CT images of tumor-bearing mice after intravenously injection of Bi_2_S_3_-3BP at different time points.
